# Intense community dynamics in the pre-Roman frontier site of Fermo (ninth–fifth century BCE, Marche, central Italy) inferred from isotopic data 

**DOI:** 10.1038/s41598-023-29466-3

**Published:** 2023-03-03

**Authors:** Carmen Esposito, Melania Gigante, Federico Lugli, Pasquale Miranda, Claudio Cavazzuti, Alessandra Sperduti, Marco Pacciarelli, Simon Stoddart, Paula Reimer, Caroline Malone, Luca Bondioli, Wolfgang Müller

**Affiliations:** 1 School of History, Archaeology and Religion, Cardiff University, Cardiff, CF10 3EU UK; 2 School of Natural and Built Environment, Queen’s University Belfast, Belfast, BT7 1NN UK; 3 Department of Cultural Heritage, University of Padua, 35139 Padua, Italy; 4 Laboratory of Osteoarchaeology and Paleoanthropology (Bones Lab), Department of Cultural Heritage, University of Bologna, 48100 Ravenna, Italy; 5 Department of Chemical and Geological Sciences, University of Modena and Reggio Emilia, 41125 Modena, Italy; 6 Department of Humanistic Studies, University of Naples Federico II, 80133 Naples, Italy; 7 Department of History Cultures Civilizations, University of Bologna, 40124 Bologna, Italy; 8 Bioarchaeology Service, Museum of Civilizations, 00144 Rome, Italy; 9 Department of Asian, African and Mediterranean Studies, University of Naples “L’Orientale”, 80134 Naples, Italy; 10 Department of Archaeology, University of Cambridge, Cambridge, CB2 3ER UK; 11 Institute of Geosciences, Goethe University, 60438 Frankfurt, Frankfurt Am Main, Germany; 12 Frankfurt Isotope and Element Research Center (FIERCE), Goethe University, 60438 Frankfurt, Frankfurt am Main, Germany

**Keywords:** Biogeochemistry, Archaeology, Biological anthropology

## Abstract

The Early Iron Age in Italy (end of the tenth to the eighth century BCE) was characterized by profound changes which influenced the subsequent political and cultural scenario in the peninsula. At the end of this period people from the eastern Mediterranean (e.g. Phoenicians and Greek people) settled along the Italian, Sardinian and Sicilian coasts. Among local populations, the so-called Villanovan culture group—mainly located on the Tyrrhenian side of central Italy and in the southern Po plain—stood out since the beginning for the extent of their geographical expansion across the peninsula and their leading position in the interaction with diverse groups. The community of Fermo (ninth–fifth century BCE), related to the Villanovan groups but located in the Picene area (Marche), is a model example of these population dynamics. This study integrates archaeological, osteological, carbon (δ^13^C), nitrogen (δ^15^N) (n = 25 human) and strontium (^87^Sr/^86^Sr) isotope data (n = 54 human, n = 11 baseline samples) to explore human mobility through Fermo funerary contexts. The combination of these different sources enabled us to confirm the presence of non-local individuals and gain insight into community connectivity dynamics in Early Iron Age Italian frontier sites. This research contributes to one of the leading historical questions of Italian development in the first millennium BCE.

## Introduction

**Figure 1:**
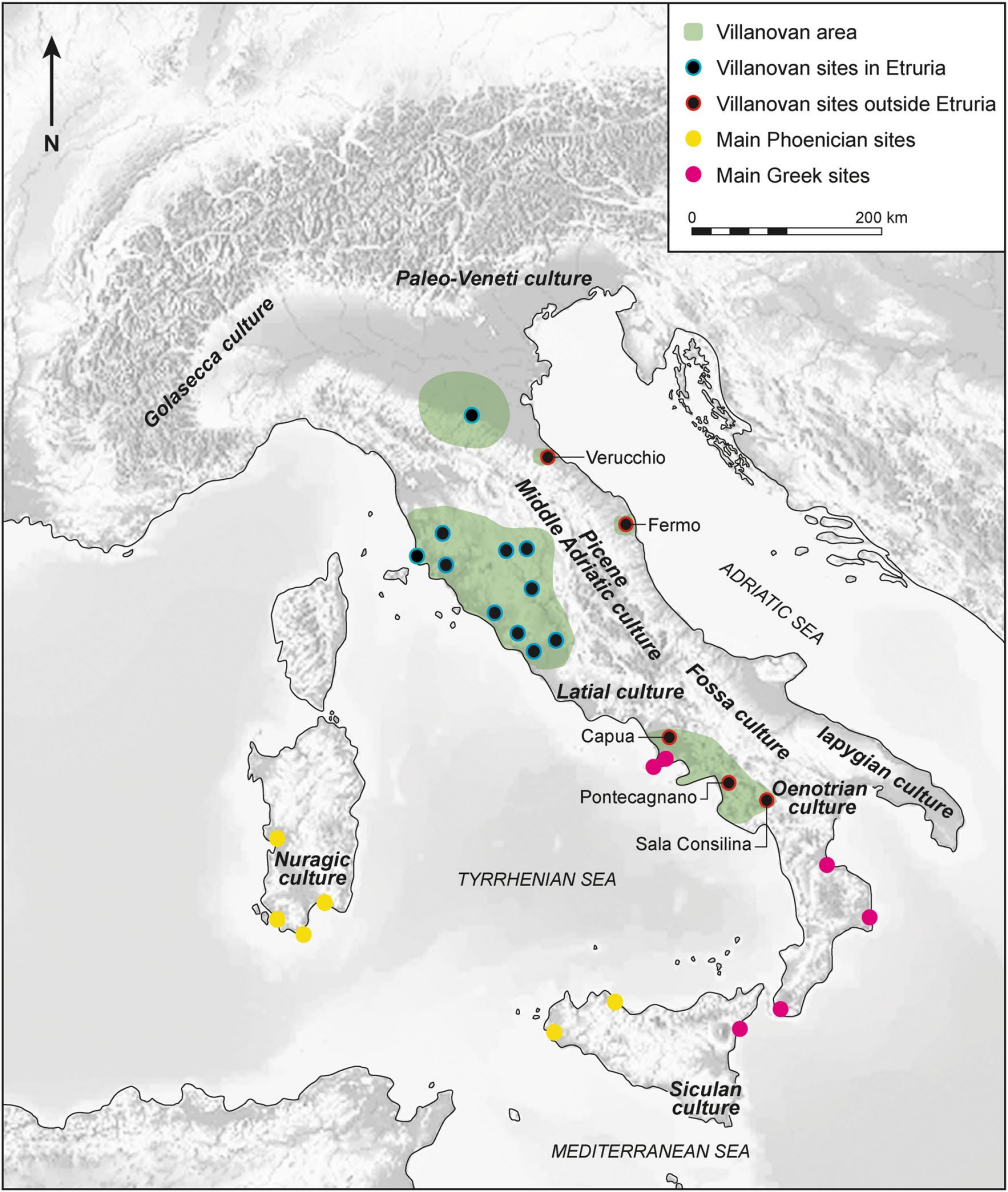
Italy during the Early Iron Age (tenth–eighth century BCE). Diverse cultural groups are archaeologically identified in the Italian Peninsula, Sicily and Sardinia. Green area: Villanovan areas; black dot with blue border: some of the main Villanovan sites; black dot with red border: Villanovan sites outside Etruria; pink dot: some of the main Greek sites; yellow dot: some of the main Phoenician sites. The open access map was downloaded from (https://pixabay.com/illustrations/italy-alps-alpine-region-map-1804893/) and modified in Adobe Illustrator 2023 27.1.1 (Adobe Inc.).

Important changes characterized the Italian Peninsula during the Early Iron Age (EIA: end of the tenth to the eighth century BCE)^1^. After the Final Bronze Age, most cultural entities experienced profound social, cultural and structural internal changes associated with the increase of exchange networks among local and incoming groups. In this period, archaeological assemblages, defined by distinctive material culture and funerary rituals—which arguably corresponded later to specific ethnic entities (e.g. Latial culture = Latins, *Atestina* culture = *Veneti*, Golasecca culture = *Leponti*)—emerged regionally across the Italian Peninsula^1^. Meanwhile, in a later phase Phoenicians and Greek people from the eastern Mediterranean started to settle in Italy, Sicily and Sardinia^2^. The human groups associated with the Villanovan material culture (tenth–eighth century BCE) stood out among the emerging local groups because of their structural and territorial organization and dominant presence within a large part of Italy^3^. The main concentration of Villanovan sites was in Etruria in the central Tyrrhenian region and in the southern Po plain. Other sites, such as Verucchio, Pontecagnano, Capua, Sala Consilina and Fermo—the latter being the focus of this work—were scattered across the peninsula outside the main Villanovan area. Some scholars have recognized these sites as the outcome of the Villanovan (also known as Proto-Etruscan) expansion^4^, while others have favoured the interpretation of a local formation^5,6^ (Fig. 1).

The Villanovan material and ritual practices have been considered representative of one cultural entity because of the recurrent features and the geographical and cultural continuity between Villanovan and Etruscan settlements and necropolises^7^ (seventh–first century BCE), where the Etruscans have been considered an ethnic group recognized in the written sources. Villanovan sites often show: (a) a highly formalized funerary cremation ritual where typical vases with a distinctive neck were covered by in-turned rim bowls—both decorated with a distinctive geometric style—and employed as containers of the cremated human remains^8^; the urn was then placed within a pit and filled with “*terra di rogo*”—the pyre ashes (Supplementary Fig. S1); (b) a unique settlement structure which consisted of well-defined vast plateaux or hilltops (120–200 hectares, while secondary sites were 25–90 hectares) surrounded by necropolises and interpreted as a form of early state formation^9^; and (c) diagnostic bronze artefacts (e.g. bronze helmets, disc fibulae) which recurred in Villanovan necropolises.

Villanovan sites in Etruria witnessed a rapid and marked process of demographic nucleation together with a process of identity formation^10^. At the same time, the sites within and outside Etruria interwove relationships with different local populations and later with Greek people, as indicated by the presence of artefacts from different sources^4^ and hybrid features in the funerary ritual made up of mixed local and Villanovan funerary traditions^11^. The mixed assemblages documented in Villanovan sites outside Etruria have provoked vibrant debates about the ethnicity of those places^6,12^. Consequently, greater effort has been invested in the ethnic classification of those sites, neglecting aspects related to human connectivity and the impact the former had on the Villanovan group as a whole (see Supplementary Note).

### The archaeological nature of the Fermo site

The funerary context of Fermo consists of the Misericordia (ninth–seventh century BCE) and the Mossa (second half of the eighth century BCE to the early fifth century BCE) necropolises, located along the north-west and north-east slopes of the Girfalco hill (Supplementary Fig. S2) in the central part of the Picene territory, on the Adriatic coast in the area between the Foglia and Tronto rivers (in the modern Marche region). Several excavations conducted at Fermo from the beginning of the twentieth century brought to light approximately 345 tombs, including both cremations and inhumations (see Supplementary Table S1 for the excavation campaigns). As recently suggested by Miranda and Esposito the two necropolises were used over five centuries (i.e. ninth to the early fifth century BCE), based on the typological analysis of the artefacts which has identified five main phases (I–V)^13^. In the current study, tombs from the ninth to the sixth century BCE were analysed and classified in three main chronological phases to ensure that each phase was adequately represented: Phase A, from the second half of the ninth century BCE to the first half of the eighth century BCE (represented by Phases I and IIA in Miranda and Esposito^13^); Phase B, the second half of the eighth century BCE (represented by Phase IIB in Miranda and Esposito^13^); and Phase C, from the seventh to the sixth century BCE (represented by Phases III and IV in Miranda and Esposito^13^). Phase V (end of the sixt to the beginning of the fifth century BCE) was not included in this study since the skeletal series from this phase is still under examination. Radiocarbon dating of inhumed and cremated bone samples from different phases as well as grave goods will be presented elsewhere because a long discussion—not possible here—of some methodological aspects is necessary.

**Figure 2:**
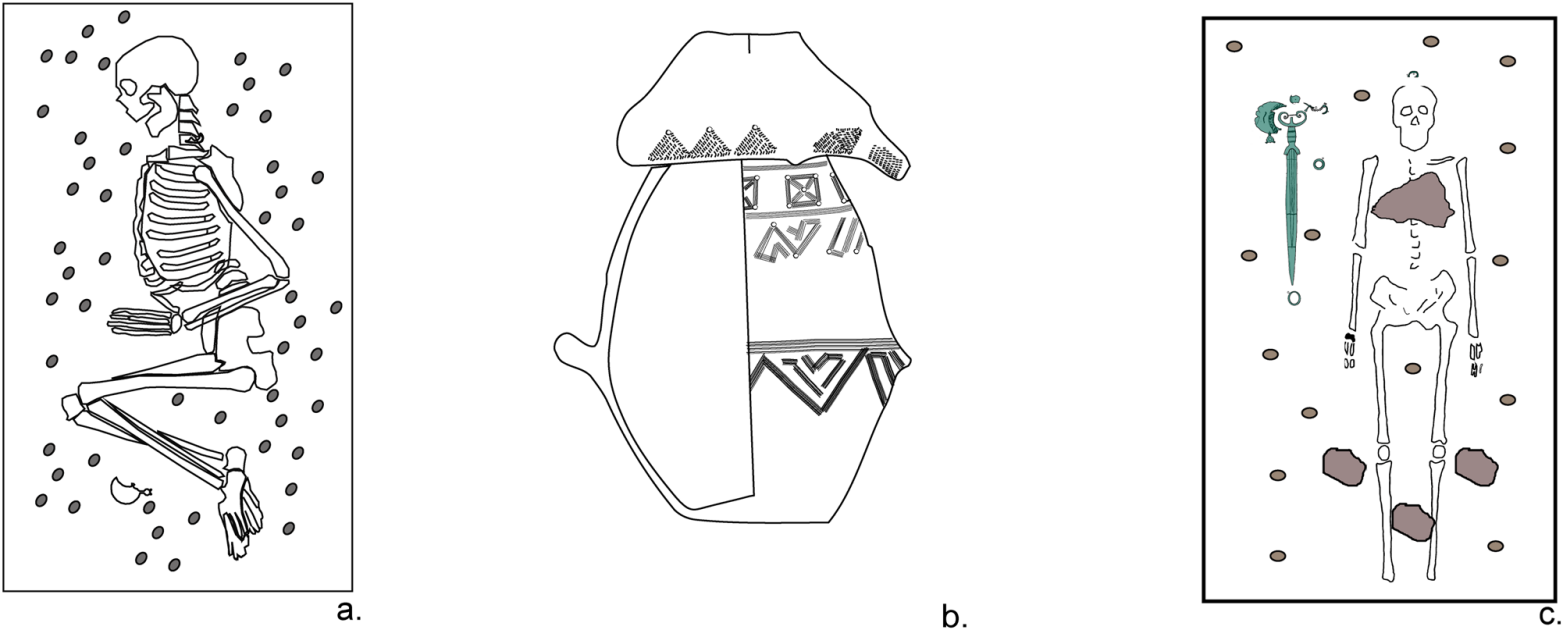
Typical Picene funerary and Fermo funerary traditions. (**a**) Picene burial, Tomb AI18, Porto Sant’Elpidio, adapted from Bergonzi and Ritrecina^64^; (**b**) a vase with a distinctive neck covered by a bowl containing human cremation, Tomb 13, Fermo, Misericordia necropolis (adapted from a drawing of the Soprintendenza Archeologia, Belle Arti e Paesaggio delle Marche), Alidori excavation; (**c**) supine inhumation on gravel bed, Tomb 19, Fermo, Misericordia necropolis, Brusadin excavation.

The material culture and funerary ritual at Fermo reflected a completely different cultural profile compared with the surrounding Picene necropolises, especially in the earliest phases^14^. Fermo is itself located within a territory spread across the Marche and northern Abruzzo regions, characterized by the Picene material culture^15^. This archaeological culture is identified by the funerary ritual, which consisted of inhumations where crouched bodies were often placed on gravel beds (Fig. 2a), and by ornaments typical of this culture, such as spectacle fibulae, zoomorphic pectorals, bronze *falere* and the *kothon* (generally associated with female grave goods), a vase likely of ritual use^15^. Finally, the Picene area overall displays a different territorial organization compared with the contemporary Villanovan area of Etruria. Indeed, some studies have suggested that settlements in the medio-Adriatic area reached up a magnitude of roughly 20/30 hectares during the EIA1 (tenth to ninth century BCE)^16^. This is comparable to the proto-urban phenomenon of *Latium vetus* represented by sites that are smaller in scale compared to the ones in Etruria. However, it is not possible to recognize for the medio-Adriatic area a proto-urban phenomenon similar to the one attested in the Tyrrhenian sites due to a lack of necropolises that consist of hundreds or at least many tens of burials, like those of Etruria and *Latium vetus* during the EIA1. This might be due to a gap in the archaeological research, but it cannot be ruled out that the population size of the Picene centres grew rather slowly, increasing only later in the EIA2 (ninth to eighth century BCE).

Conversely, the funerary ritual at Fermo also includes the contemporary presence of cremations in Villanovan-type distinct-neck vases^17^ covered by bowls (Fig. 2b), concentrated in Phases A and B, along with supine inhumations only rarely laid out on gravel beds (Fig. 2c), documented in all chronological phases. In the earliest phases (Phase A), the ritual code in Fermo as in other Villanovan necropolises is quite specific and rigid, while a reworking of the symbols and codes is attested later on (Phase B). Moreover, the supposed expansion of the Fermo settlement (100 hectares according to Peroni^5^, but see Naso^18^, who estimates approximately 38 hectares), together with the position of the two necropolises, recall the smaller proto-urban model of Etruria. The presence of Villanovan funerary practices (although with some limited Picene influence), material culture and settlement structure has suggested Fermo as a Villanovan “island”^19^, “outpost”^20^ or “colony”^21^ in the Picene territory.

This work contributes to the understanding of the community structure of Fermo, by estimating the incidence of people born elsewhere over the course of time, using for the first time in this funerary context carbon (δ^13^C), nitrogen (δ^15^N) and strontium (^87^Sr/^86^Sr) isotope analyses (see Supplementary Note) on a sample of human bones and teeth, associated with archaeological, (palaeo)environmental and human osteological data. The present study examines the mode and tempo of residential mobilities, providing information on aspects otherwise invisible through the archaeological record alone, thus attempting to answer the long-standing research questions on the nature of Villanovan sites outside Etruria. Indeed, this article sets out to make a major contribution to one of the leading historical questions of Italian development in the first millennium BCE.

## Results

### Morphological and morphometric analyses of the human remains at Fermo

Out of 72 tombs (38 Misericordia necropolis; 34 Mossa necropolis) the osteological analysis identifies 120 individuals (Supplementary Table S2). Seventy-three individuals were inhumed (I), while 47 individuals were cremated (C), unevenly distributed among the three chronological phases (A, B and C) (Supplementary Table S3).

Of the sample so far analysed, the Misericordia necropolis had the highest percentage of cremations, reaching 93.3% in Phase A and 64.3% in Phase B; Phase C is represented in the analysed sample by a single inhumation grave only. The Mossa necropolis was not used during Phase A; cremations represented 41.4% of the burials in Mossa’s Phase B, and disappeared in Mossa’s Phase C.

Tombs contained single (n = 52), double (n = 11) or collective (n = 9) burials (the latter ranging from 3 to 14 individuals). At Misericordia, tombs were 89.5% single, 10.5% double, but collective tombs were not present. At Mossa, tombs were 52.9% single, 20.6% double and 26.5% collective. Moreover, double or collective burials were documented exclusively in the latter two phases, namely from the end of the eighth century BCE. The osteological analysis has estimated the presence of 34 females (F = 26; F(?) = 8), 48 males (M = 38; M(?) = 10), 13 adults of indeterminate sex (IND) and 25 subadults (SA; 0–15 years of age). Subadults include 2 newborn/early infants (NI = 0–1 years of age), 10 young children (YC = 1–5 years of age), 10 older children (OC = 5–10 years of age) and 3 juveniles (J = 10–15 years of age). Sex and age-at-death did not influence the choice of the funerary ritual: individuals of all age classes were cremated or inhumed. The sex ratio (M/F) for cremated individuals is 0.9. By contrast, the M/F ratio is less balanced for inhumed individuals, namely 2.2. The M/F ratio by chronological phase is: Phase A = 0.4; Phase B = 1.6; Phase C = 2.1.

### δ^13^C_VPDB_ and δ^15^N_AIR_ results

**Figure 3:**
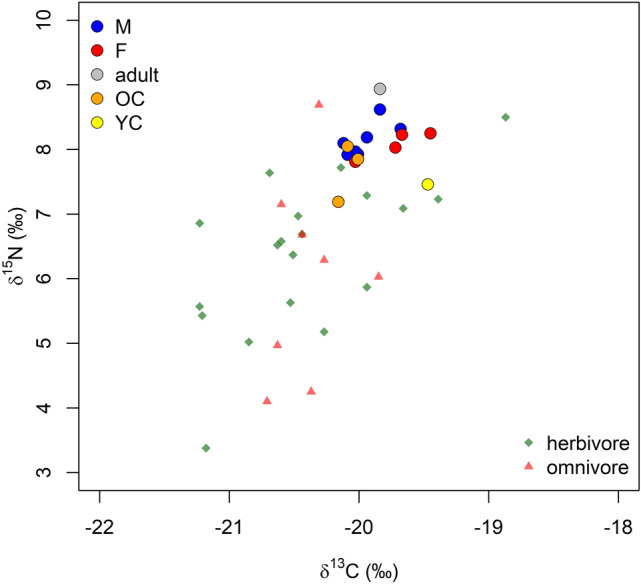
Fermo δ^13^C and δ^15^N result values for human samples according to sex and age-at-death. δ^13^C and δ^15^N baseline values (i.e. omnivores and herbivores) derive from palaeo(environmental) studies conducted on the closest available site to Fermo that is known in the scholarly literature, namely Coppa Nevigata^23^. Yellow dots: young children (YC = 1–5 years of age); orange dots: older children (OC = 5–10 years of age); blue dots: adult males (M, M(?)); red dots: adult females (F, F(?)); grey dots: adults indeterminate.

Nine out of 25 samples did not meet quality control criteria (Supplementary Table S4) and were excluded from the analysis. For the remaining 16 human samples, C:N atomic ratios (C:N_a_) fall in the range between 2.9 and 3.6, which indicates good collagen preservation^22^. Female individuals (n = 4) have δ^13^C that ranges from − 20.03 to − 19.45 ‰ and δ^15^N from 7.81 to 8.25 ‰. Male individuals (n = 7) have δ^13^C that has a range from − 20.12 to − 19.68 ‰ and δ^15^N from 7.92 ‰ to 8.62 ‰, while for only one adult individual sex was not determined (δ^13^C = − 19.84 ‰; δ^15^N = 8.94 ‰). Subadult individuals (n = 4) (all outside the breastfeeding period) have δ^13^C that ranges from − 20.16 to − 19.47 ‰ and δ^15^N from 7.19 to 8.05 ‰. While the δ^13^C range is overall similar for all the analysed categories, δ^15^N is slightly higher for male individuals, but these are marginally more represented than the other categories. Since faunal remains are poorly represented at Fermo, the environmental context has been estimated based on a study conducted on the closest available site to Fermo^23^ (Supplementary Table S5). Even though the selected samples come from different chronological and geographical environments, these represent the best available comparative record. δ^13^C and δ^15^N values for Fermo human samples are here plotted together with faunal isotope values from a Bronze Age site in Puglia (Fig. 3), to our knowledge, the single comparative sample available in the literature for the Adriatic region and closer in time to Fermo. The individuals from Fermo had a diet based on C3 plants and displayed an extraordinarily narrow variability, thus suggesting a homogenous diet across all sexes and ages. Additionally, these data exclude the significant consumption of marine food among Fermo’s individuals, important information for the ^87^Sr/^86^Sr results (Supplementary Note).

### ^87^Sr/^86^Sr results

#### Geological assessment of the area around Fermo

The area around Fermo, located between the coast and the Apennines, is mainly dominated by sedimentary sequences—marly limestone and sandstone—which comprise Upper Cenozoic (Pliocene–Pleistocene) formations with ^87^Sr/^86^Sr values ranging from 0.7085 to 0.7092^24^. A mineral water sample from the area (43°09′00.00″ N 13°43′12.00″E) has produced a ^87^Sr/^86^Sr value of 0.7090^25^ (Supplementary Fig. S3).

### Biologically available strontium (BASr) at Fermo

Understanding bioavailable strontium through careful analysis is essential for interpreting human mobility^26^; the baseline samples collected at Fermo (archaeological fauna tooth enamel n = 2; snail shell n = 2; soil n = 1) have ^87^Sr/^86^Sr values in the range of 0.7085 to 0.7088, which is narrower than expected for the regional geological formations. For this reason, other samples were also collected in a maximum 7 km radius (soil n = 2; grass n = 2; water n = 2), yielding an ^87^Sr/^86^Sr range of 0.7087 to 0.7092 in agreement with the geological data (see Supplementary Table S6 and Supplementary Fig. S4). Snail shells from the Mossa necropolis display less radiogenic values than the other samples (FM-SR-SNAIL2 = 0.7085, FM-SR-SNAIL3 = 0.7086). The overall BASr range estimated for Fermo is 0.7085 to 0.7092.

### Strontium results from human samples at Fermo

The list of ^87^Sr/^86^Sr values for 54 human individuals analysed at Fermo is provided in Supplementary Table S7. The corresponding ^87^Sr/^86^Sr ratios of human samples range between 0.70882 and 0.70968, which is wider than expected from the local geological formation and BASr values. Some basic statistical parameters on the entire sample are reported in Supplementary Table S8. The complete dataset has a mean of 0.70900 and has a rather narrow standard deviation (sd = 0.00019).

## Discussion

The local ^87^Sr/^86^Sr baseline is based on a cumulative approach (see Methods and Supplementary Note for details), namely: (a) an initial geological assessment of the area around Fermo (see ^87^Sr/^86^Sr results); (b) biologically available strontium (BASr) on modern environmental samples and archaeological specimens (see ^87^Sr/^86^Sr results); (c) statistical analysis of the shape of the human sample distribution; and (d) the range of values for the youngest individuals in the sample, namely young children (YC = 0–5 years of age) and older children (OC = 5–10 years of age). As reported in the results, diverse samples (e.g. archaeological fauna, snail shell, soil, grass and water) were collected within 7 km of Fermo. In agreement with other studies^24^, snail shell samples have lower ^87^Sr/^86^Sr values, and this might be due to the amount of soil carbonate incorporated in their diet^27^. Therefore, even if snail shells have been used to construct local baselines^28,29^, they seem not to be the most appropriate samples in the Fermo case. By contrast, grass (FM-SR-GRASS1 = 0.7091, FM-SR-GRASS3 = 0.7092) and water (FM-SR-WATER1 = 0.7091, FM-SR-WATER2 = 0.7091) have higher values than soil and archaeological fauna (Supplementary Fig. S4). A possible explanation is that sea spray (marine ^87^Sr/^86^Sr = 0.7092—McArthur et al.^30^) and/or dust influenced grass and, to a certain extent, water values in the area between 0 and 7 km distance from Fermo^31^. To make a good estimate of the BASr at Fermo, snail shells and grass values are excluded as too low and too high respectively. Hence, the BASr for Fermo is considered to range between 0.7087 and 0.7091.

If we consider the overall ^87^Sr/^86^Sr human data (c), the normal probability quantile–quantile plot (Q–Q plot) of human samples ^87^Sr/^86^Sr values (Supplementary Fig. S5) graphically highlights at least 9 out of 54 samples that visibly stand out from the normal distribution. The central trend of the human data at Fermo, which is given by our entire sample except the 9 outliers identified in the Q–Q plot, ranges from 0.70882 to 0.70909.

The shape of the sample distribution of ^87^Sr/^86^Sr helps identify possible outliers and age-related patterns. The kernel density estimate of the sample distribution (Supplementary Fig. S6) shows a multimodal pattern with a skewed tail towards higher radiogenic values. Using the young and older children’s (d) intervals as possible additional proxies of the local signal (see Supplementary Note), it is possible to define a local range of 0.70883 to 0.70908 that matches the shape of the whole sample distribution. Using Tukey’s interquartile range method (1.5xIQR) to identify outliers^32^, only 4 individuals showing the highest radiogenic values can be considered outliers.

Hence the various baseline ranges, namely: environmental values (0.7087 to 0.7091), the shape of the sample distribution of the human data (0.70882 to 0.70909) and the values based on the youngest individuals in the sample (0.70883 to 0.70908) are overall in good agreement, while Tukey’s IQR method is evidently too broad and tends to include individuals outside the normal distribution of the ^87^Sr/^86^Sr ratio.

If the wider baseline range (0.7087 to 0.7091) is considered as the most probable interval to identify individuals born locally, regardless of consideration of differential chronology and ritual, the number of non-local individuals at Fermo is 9 (16.7%), with individuals with values between 0.7091 and 0.7092 possibly considered as non-locals and those higher than 0.7092 as non-locals.

The origin and character of the Villanovan sites outside Etruria have been the subject of debate for a long time. However, the nature of the archaeological record did not always allow a clear-cut interpretation of the sites (see Supplementary Note). The δ^13^C and δ^15^N data at Fermo illustrates that overall, inhumed individuals had a relatively homogenous diet, consuming C3 plants and a reasonable amount of protein with no or negligible marine food consumption. This latter aspect is crucial for a correct evaluation and assessment of ^87^Sr/^86^Sr analysis since marine strontium values (0.7092) can influence general ^87^Sr/^86^Sr values. Furthermore, the combined findings of the current study indicate for the first time the presence of non-local individuals at Fermo, disproving the hypothesis of a completely local formation of the site (see Supplementary Note). The percentage of 16.7% of non-local individuals is higher than in the contemporary Iron Age contexts (ca 8%)^33^. However, this general figure must be unravelled in the light of osteological (sex and age-at-death) and archaeological (chronology and funerary ritual) information.

**Figure 4:**
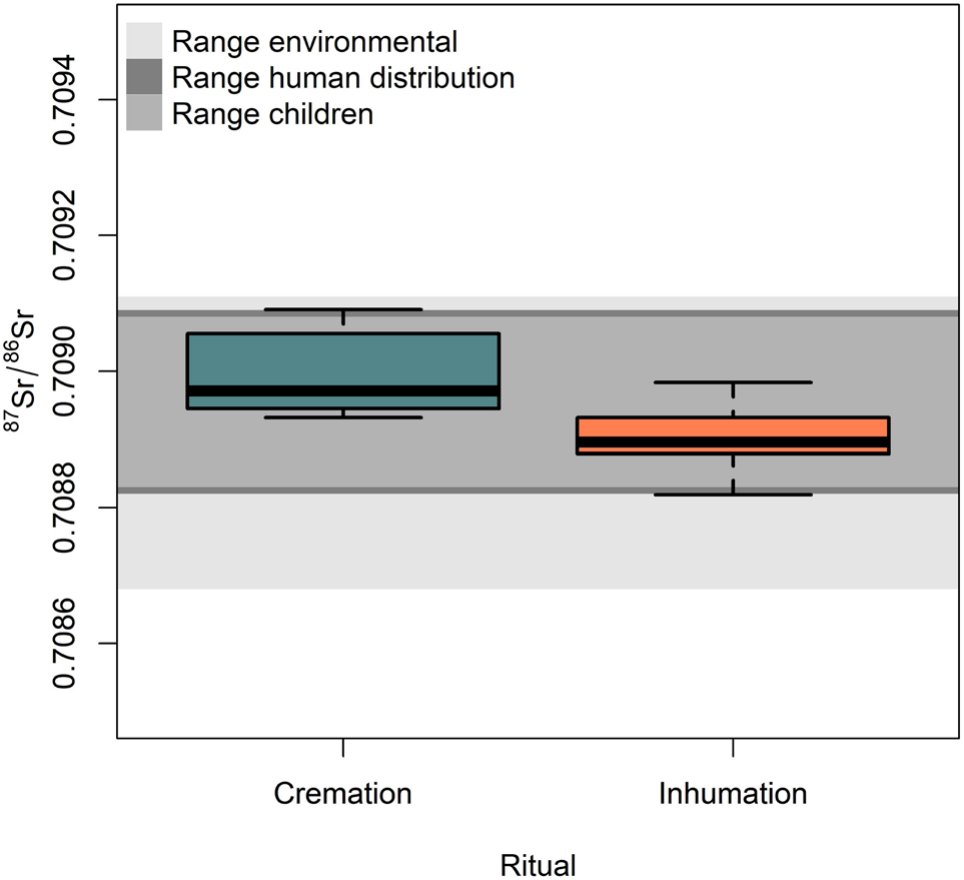
Box–and–whiskers plot of ^87^Sr/^86^Sr range for cremations (petrous bone and tooth enamel) and inhumations (tooth enamel) at Fermo with the three baseline ranges (i.e., environmental samples, human distribution, children range) in grey, considering only individuals that fell within the three baselines.

Even though diverse types of samples were used for cremated (*pars petrosa* and more rarely tooth enamel) and inhumed (tooth enamel) individuals, the ^87^Sr/^86^Sr values for the two mortuary practices display a similar range (cremation: min = 0.70893 and max = 0.70968; inhumation: min = 0.70882 and max = 0.70958) in the entire sample. Conversely, if we consider only the individuals that fell within the wider baseline range, the difference between the ^87^Sr/^86^Sr range in cremations and inhumations is statistically significant (t = 3.8362, df = 37.564, *p*-value = 0.0002). Figure 4 illustrates that ^87^Sr/^86^Sr values in cremated individuals are more radiogenic than inhumed ones even if they partially overlap (cremation: min = 0.70893 and max = 0.70909; inhumation: min = 0.70882 and max = 0.70898). Tooth enamel samples’ ^87^Sr/^86^Sr values are less radiogenic and closer to the ^87^Sr/^86^Sr values of environmental samples collected at the site of Fermo, namely soil and fauna samples (^87^Sr/^86^Sr = 0.7087).

Hence, it could be hypothesized that tooth enamel ^87^Sr/^86^Sr values were overprinted by diagenesis^34^. However, this hypothesis is weak because of the well-known resistance of enamel to diagenesis^35^ and the fact that dentine was completely removed from the tooth thanks to a specific sampling protocol (see Methods and Supplementary Fig. S7). By contrast, it is unlikely that the petrous bone from cremated individuals underwent diagenesis^36^ because the samples were fully calcined, as shown by FTIR (Fourier Transform Infrared Spectroscopy) analysis run for ^14^C dating (see Supplementary Table S7). It can arguably be suggested that the difference in ^87^Sr/^86^Sr values in cremated and inhumed individuals is likely related to endogenous factors. Therefore, we detail the analysis of the Fermo samples, considering cremated and inhumed remains separately.

**Figure 5:**
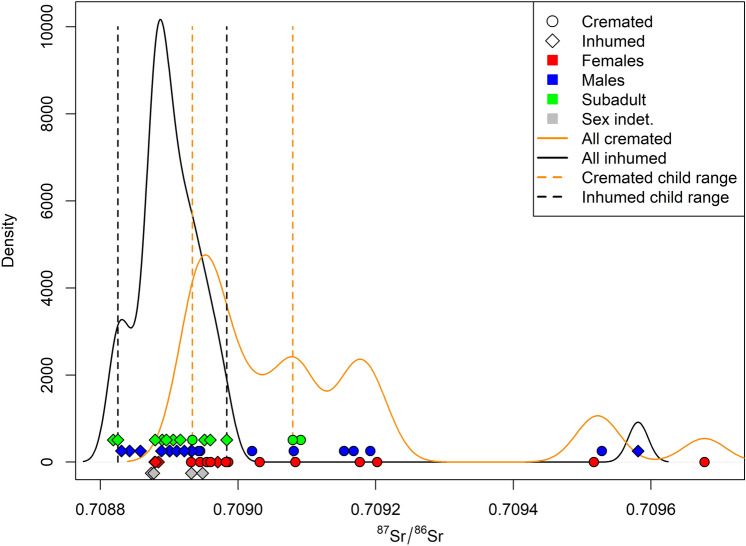
Density plot of cremated and inhumed ^87^Sr/^86^Sr values for Fermo according to sex and age-at-death. Dots: cremated individuals; diamonds: inhumed individuals; red: adult females (F, F(?)); blue: adult males (M, M(?)); green: subadults (YC, OC, J); grey: adult indeterminate; orange dotted lines: cremated children range (0.70893–0.70908); black dotted lines: inhumed children range (0.70883–0.70898).

Figure 5 illustrates cremated and inhumed individuals distributed over two kernel density plots. The inhumed density plot is more homogenous, while the cremated one is broader and tends towards more radiogenic values. The children range for inhumed and cremated individuals partially overlap, but a clear tendency toward more radiogenic values is evident for cremated children too. According to this evidence, it is possible to suggest that the range of inhumed children might represent a more reasonable estimate of the local range at Fermo and indicate that some of the cremated children could have been born elsewhere. In any case, the entire range for children (0.70883 to 0.70908) can be assumed as a more parsimonious estimate of the local range. If we adopt this latter as the ^87^Sr/^86^Sr baseline at Fermo, the number of non-local individuals rises to 12 (22.2%), considering that the lower value of the entire children range (0.70883) lies within the FM-SR-38 = 0.70882 ± 0.00002 interval (see Supplementary Table S7), considering this latter as local.

Among cremated individuals (n = 24), 45.8% are non-local, while 3.3% of the inhumed individuals are allochthonous. Notably, the mobility seems similar among male and female individuals (5 and 6 individuals respectively), thus adding new insight about the biological sex of individuals who moved to Fermo. The sex ratio (Phase A + Phase B) is 1.1, and therefore it does not bias the scoring of mobility by sex.

**Figure 6:**
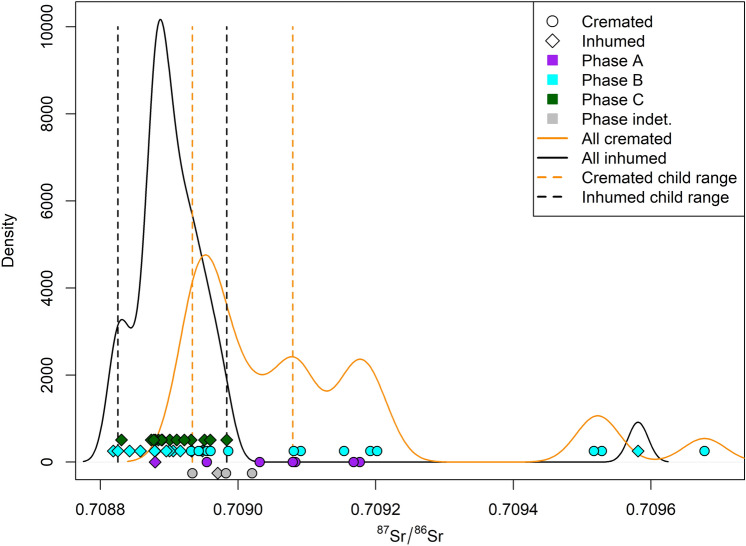
Density plot of cremated and inhumed ^87^Sr/^86^Sr values for Fermo according to chronological phases. Dots: cremated individuals; diamonds: inhumed individuals; purple: Phase A (end of the ninth century to the beginning of the eighth century BCE); light blue: Phase B (second half of the eighth century BCE); green: Phase C (seventh and sixth century BCE); grey: indeterminate chronology; orange dotted lines: cremated children range (0.70893–0.70908); black dotted lines: inhumed children range (0.70883–0.70898).

If the samples are considered according to chronological phases (Fig. 6), 3 out of 7 individuals from Phase A are non-local. Even if the small sample number for Phase A requires a cautious approach, a substantial percentage of individuals (42.9%) can be considered non-local. Nine individuals out of 29 from Phase B are non-local, namely 31%. All the individuals from Phase C fall within the children range; hence they can all be considered locals. The 4 individuals with indeterminate chronology are all local.

**Figure 7:**
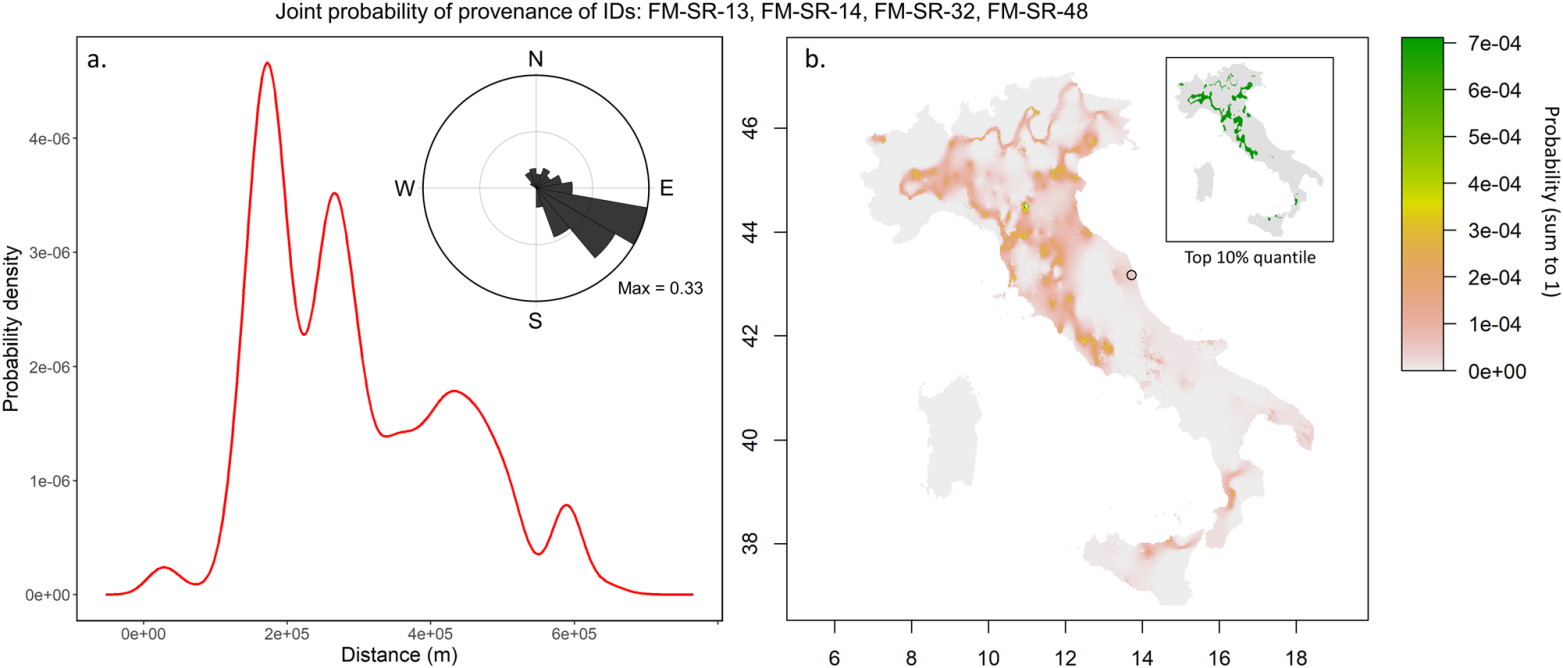
(**a**) Probability density plot representing the likely distance of movements for the most radiogenic non-local individuals (FM-SR-13, FM-SR-14, FM-SR-32, FM-SR-48; ^87^Sr/^86^Sr = (0.70952–0.70968)) from their hypothetical location of origin (see panel b) to reach Fermo. The distribution is multimodal with a peak at ~ 180 km, i.e. the most likely distance travelled by these individuals. In the inset, the bearings travel distribution shows the more likely path taken by the non-local individuals to reach Fermo. These values are obtained comparing the likely geographical origin of the sample (as depicted in panel b) and the site location. (**b**) Joint probability density surface showing the likelihood of geographical provenances for the non-local individuals. Each cell’s value represents the probability that the specific cell is the actual origin of the sample (green = highest probability, white = lowest probability, square cell side length = 2.5 km). The probabilities sum to 1. These analyses were performed in R, using the “assignR” package by Ma et al.^37^. The provenancing is based on the Universal Kriging model of the Italian Sr isoscape presented in Lugli et al.^24^. The map is built in R version 4.0.5 (see the code in the Supplementary Note; https://www.r-project.org/).

Figure 7 reports a tentative application of the Ma et al.^37^ method for isotope-based geographical assignment. The method itself attempts to infer the geographical origin from an isoscape based on a variety of isotopes. Although the present analysis employs a single isotope isoscape^24^ and the stochastic approach of the method itself, it is worth noting that results fit nicely with the archaeological expectation of the most radiogenic individuals probably originating ca. 180 km away from Fermo within an area that encompasses the original Villanovan area of Etruria.

The current study has, by comparing archaeological (i.e. chronological and funerary ritual information) and isotopic data, revealed for the first time that people arrived at the site at different times, even if new arrivals decreased from Phase A to Phase C. Moreover, the high percentage of non-locally born individuals, albeit on a small sample, in Phase A is consistent with the archaeological data^38^; indeed, a rigorous ritual tradition which consists of few grave goods that accompany traditional distinct-neck urns that strictly followed models from Etruria was present at Fermo in the earliest phases. The presence of allochthonous individuals in Phase B adds to the previous archaeological hypothesis^19–21^ that suggests human mobility at Fermo only in the initial phase, namely the ninth century BCE. Finally, the absence of non-local individuals in Phase C is in line with more recent archaeological interpretations, which have shown that Fermo developed its own funerary practice and autonomous tradition in this period, which differs from typical late Villanovan/Etruscan and Picene customs^13^. A possible reconstructed scenario could be that a group of at least partially non-local individuals who settled at Fermo (Phase A) were followed by other individuals coming from the original homeland (Phase B). Later, this flow of non-local individuals ended (Phase C), and Fermo developed a peculiar local mixed funerary tradition.

The isotopic results have, in part, confirmed that the difference in the funerary ritual indicates a difference in the provenance of the individuals. However, it cannot be excluded that among the individuals considered isotopically local, there could be some non-local individuals from a geographical region with similar ^87^Sr/^86^Sr baseline values to Fermo. In any case, it is impossible to distinguish whether cremated individuals with local ^87^Sr/^86^Sr signatures were the offspring of immigrants or of local people who partially adopted a foreign tradition.

Some of the tombs with typical Villanovan features are isotopically local (e.g. Tomb 45, Misericordia necropolis, Bonfigli excavation). In contrast, other tombs with local and Villanovan elements can be either isotopically local (e.g. Tomb 44, Misericordia necropolis, Bonfigli excavation) or non-local (Tomb 78, Mossa necropolis, 1999–2000 excavation). Tomb 17 (Mossa necropolis, 1999–2000 excavation)—the only inhumation with the highest radiogenic ^87^Sr/^86^Sr values (^87^Sr/^86^Sr = 0.70958)—does not show any characteristic archaeological features that can recall either the Picene or the Villanovan environment.

Collective tombs such as Tomb 11 (Mossa necropolis, 1968 excavation) include both inhumations and cremations. In this tomb, the cremated individual (11_5) has non-local values (^87^Sr/^86^Sr = 0.70920), while the inhumed individuals (n = 3) fall within the local range (Tomb 11_2 = 0.70888, Tomb 11_3 = 0.70891, Tomb 11_4 = 0.70892). Even though kinship relationships cannot be fully reconstructed for these cases because of the presence of cremated individuals, these findings suggest a possible integration between allochthonous and autochthonous individuals at an extended group level.

This work illustrates the highly complex relationship between groups of diverse origins and specific forms of funerary ritual and material culture. As well as contributing towards the understanding of an important historical question, we can suggest that these relationships are multifaceted, since the agency of identity is a matter of social choice and not determined by biology.

**Figure 8:**
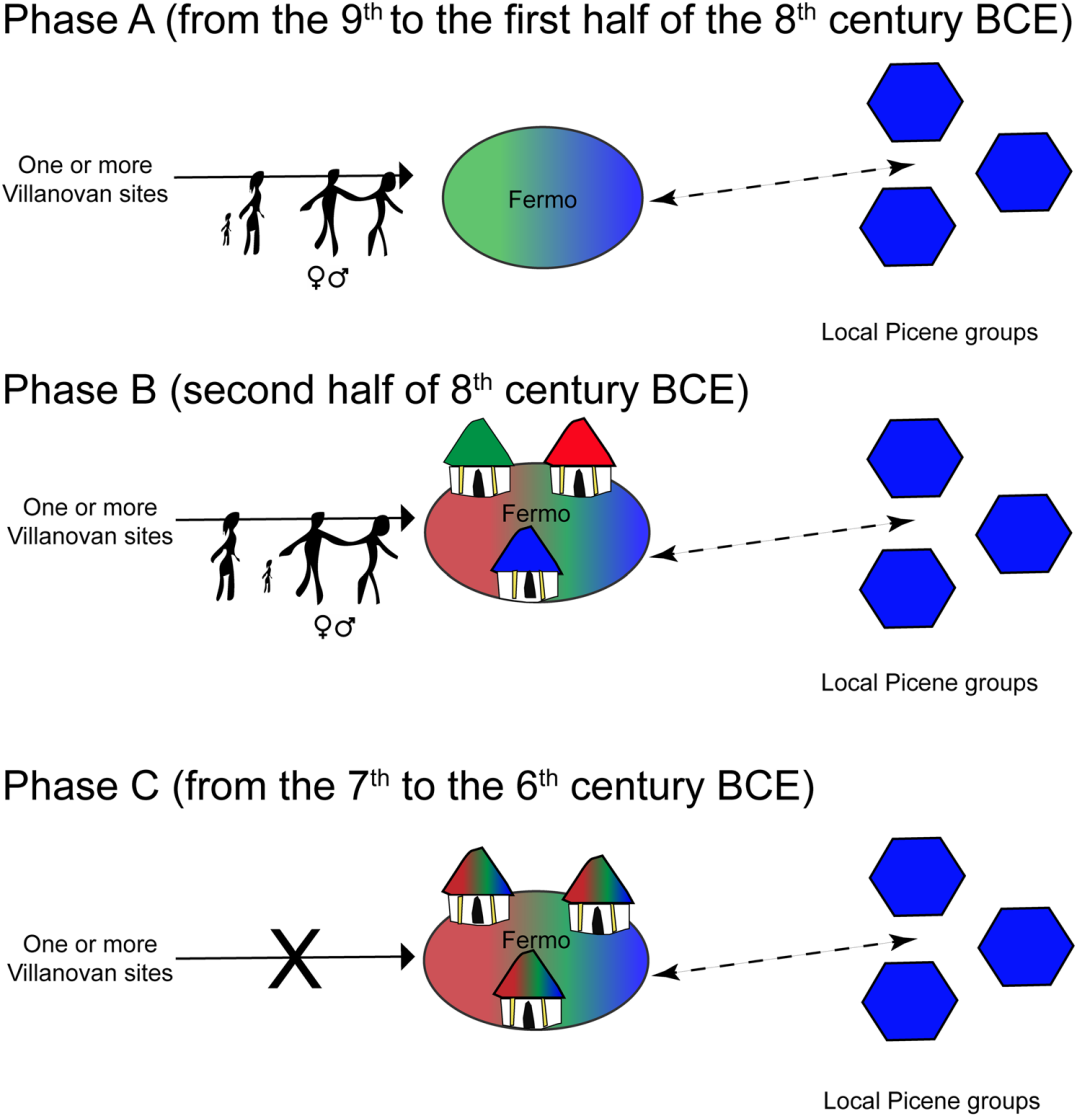
A possible reconstructive model of human mobility at Fermo. Phase A (ninth –first half of the eighth century BCE) male and female individuals arriving at Fermo from one or more Villanovan site(s); Phase B (second half of the eighth century BCE) male and female individuals arriving at Fermo from one or more Villanovan site(s); Phase C (seventh–sixth century BCE) absence of non-local individuals according to ^87^Sr/^86^Sr analysis. Black arrow: human mobility; dashed arrow: unknown relationship (based on archaeological record only).

Overall, the data highlight that groups of people of both sexes moved to Fermo in diverse periods. All the analysed data enables us to build a model for mobility phenomena at Fermo that can eventually be expanded, quantified and tested on other Villanovan sites within and outside Etruria (Fig. 8).

## Conclusion

The Italian Peninsula witnessed profound changes in the population dynamics during the EIA. The method used has for the first time provided a nuanced and multifaceted picture of the type and mode of human mobility in an EIA Italian community linked to the emerging Etruscans. From a specific archaeological perspective, the presence of non-local individuals during the first phases of life of the Fermo community has reinforced the notion that the site was part of a broader Villanovan network. The paper has indeed provided new evidence and interpretation of the changes in social construction and funerary representation that only partially emerged from the archaeological record, by directly considering aspects of human mobility from independent biological data. This work provides a model for understanding human mobility in EIA groups in Italy which can be expanded to and tested on other contemporary sites, further delineating central aspects of past human connectivity and its effect on ancient groups. It contributes to the historical reconstruction and comprehension of population dynamics and growing networks in the Italian EIA, which underwrote the later development of the Roman-world hegemony, where mobility and the capture of large populations were on a substantially greater scale.

## Methods

### Morphological and morphometric analyses of the human remains

This study analysed 72 inhumations and cremations (38 tombs from the Misericordia necropolis and 34 tombs from the Mossa necropolis). This research was accomplished following the relevant regulations for the treatment of ancient human remains. Permits for osteological and isotopic analyses were granted by the Soprintendenza Archeologia, Belle Arti e Paesaggio delle Marche and the Polo Museale delle Marche (Prot. 0000370 of 2017, Prot. 0000369 of 2017, Prot. 3556 of 2018, Prot. 3226 of 2018).

The osteological analysis focused on (a) the estimation of the minimum number of individuals (MNI); and (b) the determination of the demographic profile (sex and age-at-death) of the skeletal sample for each grave. The sex estimation of inhumed adult individuals was based on (a) dimorphic traits of the skull (e.g. nuchal crest, mastoid process, supraorbital margin, glabella and mental eminence) and pelvis (e.g. greater sciatic notch, preauricular sulcus, ischiopubic ramus); and (b) general observation of the relative robusticity/gracility of the skeleton among the skeletal series^39,40^. The sex estimation for cremated individuals was based on morphological traits and sexual metric dimorphism following Cavazzuti et al.^41^.

Age-at-death of subadults in both cremations and inhumations was based on (a) dental formation and the eruption of deciduous and permanent dentitions^42^; (b) long bone length^43^; and (c) epiphyseal closure of skeletal elements^44^. The age-at-death of adult individuals was estimated on (a) tooth-wear patterns in the permanent dentition^45^; and (b) degenerative changes of the sternal rib epiphysis^46,47^, (c) of the symphyseal surface of the pubic symphysis^48^, and (d) of the auricular surface of the ilium^49^.

### Isotopic analysis of δ^13^C_VPDB_ and δ^15^N_AIR_ in human bone collagen

To understand the dietary variability across age and sex and to estimate the marine contribution to the diet, which potentially influences the ^87^Sr/^86^Sr results, 25 inhumed individuals’ ribs were analysed for δ^13^C and δ^15^N. Collagen was extracted from the samples following the protocol of ^14^CHRONO Centre (QUB)^50^. The C:N_a_ ratio (ratio of carbon to nitrogen atoms) was determined on the same collagen by elemental analysis on the EA-IRMS^51^. All extracts that contained C:N_a_ ratios outside a range of 2.9–3.6 were not included since their isotopic composition may have been compromised by diagenetic alteration^52,53^.

### ^87^Sr/^86^Sr analysis

Overall ^87^Sr/^86^Sr methodology follows Müller et al.^54^. Fifty-four human specimens (petrous bone, n = 23; tooth enamel, n = 31) were sampled for ^87^Sr/^86^Sr isotope analyses. A balance in the ratio of the sample in accordance with funerary ritual was kept, i.e. cremations (n = 24) and inhumations (n = 30). Petrous bone samples were favoured in cremations, with only one tooth enamel (M3) sample analysed. Enamel of the first (M1, n = 27) and more rarely, second (M2, n = 1) permanent molars was used in inhumations (see Supplementary Note). Only in two cases were deciduous teeth (m1, n = 1; m2, n = 1) selected. The assessment of the local ^87^Sr/^86^Sr baseline was based on a multi-factorial approach (see Supplementary Notes), namely: (a) geological assessment of the area (Supplementary Fig. S3); (b) an estimate of the strontium isotopic ratio (^87^Sr/^86^Sr) baseline on modern environmental samples and archaeological specimens; (c) the shape of the sample distribution of the human data^55,56^, assuming that the majority of the individuals are local^57^; and (d) the range of values for the youngest individuals in the sample, namely young and older children (1–10 years of age). Regarding (b), to avoid possible bias, different samples were selected, considering the lithological compositions of the area around Fermo within a 0–7 km distance. The 11 samples included archaeological fauna from the medieval excavation of Area Vallesi at Fermo (n = 2), modern snail shells (n = 2), water (n = 2), soil (n = 3) and plants (n = 2). Multiple samples were selected from the same area to spot possible differences in ^87^Sr/^86^Sr values (see Supplementary Table S6). Woodland zones were preferred to minimize the risk of contamination by modern sources of ^87^Sr/^86^Sr (e.g. modern fertilizer). The optimum sample mass was ~ 2 g for soil, ~ 1.5 g for grass, ~ 0.015 g for snails and ~ 15 ml for water.

### Sampling procedures

As for cremations, the petrous bone was abraded to remove surface contaminants using a dental bur. The otic capsule was separated from the cremated petrous portions using a 1 mm mechanical saw mounted on a drilling machine (DREMEL® model 300), following Harvig et al.^58^. Subsequently, the densest part of the central inner ear was sampled using a low-speed drill (2 mm diameter), producing clean samples of intact otic capsules for the Sr isotope analyses. The bone powder was stored in pre-cleaned plastic Eppendorf (1.5 ml) vials. The bone powder mass ranged between 0.02 and 0.04 g. As for inhumations, upper molar enamel was sampled from the protocone, or mesiolingual, cusp to the cement enamel junction (CEJ), whereas lower molars were sampled from the occlusal margin of the protoconid, or mesiobuccal, cusp to the CEJ, following Müller et al.^54^. A flexible diamond-edged rotary wheel mounted on a drilling machine (DREMEL® model 300) was used to cut a longitudinal crown section of the cusps. Adhering contaminants such as soil, sediments and all trace of dentine were removed using a dental bur. The tooth enamel mass ranged between 0.02 and 0.04 g.

For 9 samples (from FM-SR-52 to FM-SR-60), a different methodology was adopted to sample bulk enamel, similar to the method proposed by Czermak et al.^59^ for dental roots. This technique was employed to remove any dentine trace, which can be affected by diagenesis and can influence ^87^Sr/^86^Sr values contained in enamel samples (Supplementary Note). Teeth were cleaned and photographed. Subsequently, samples were covered in Crystalbond 590 Mounting Adhesive (Aremco Products, Inc.) before being embedded in resin. Crystalbond is a transparent resin, reversible in acetone, which isolates the tooth when it is embedded in epoxy resin.

Teeth were embedded in an epoxy resin and subsequently longitudinally cut following Nava et al. ^60^. Sections were made passing through the tip of the dentine horn along the buccolingual plane. Once the cut was performed, dentine was thoroughly removed with a dental bur. The section was immersed in acetone to free the tooth from the resin (Supplementary Fig. S7).

### Laboratory procedure

#### Petrous bone powder

Bone powder samples were leached in 0.25 M acetic acid (CH_3_COOH). Leached samples were put in an ultrasonic agitation bath for 10 min and let stand for ~ 3 h. Samples were centrifuged. After removing the acetic acid with an acid-cleaned disposable pipette, samples were rinsed with Milli-Q® water. The remaining leachate was stored. After being centrifuged, Milli-Q® water was removed with disposable pipettes for each sample. Two ml of 14 M HNO_3_ was added to the vials. Samples were transferred from centrifuge vials to Teflon™ beakers for dissolution. Teflon™ beakers were previously cleaned with 14 M HNO_3_ and rinsed with Milli-Q® water. Teflon™ beakers were closed and placed on a hotplate overnight at 150 °C, then opened and put on the hotplate at 150 °C to be evaporated. This step was repeated (2–3 times) until the full dissolution of samples.

#### Tooth enamel samples (human and fauna)

Samples were pre-treated with 1 ml of acetone for 3–5 min in an ultrasonic agitation bath, followed by 1 ml of methanol (CH_3_OH) for 3–5 min in an ultrasonic agitation bath. Samples were rinsed three times, with 1 ml of Milli-Q® water, taking care of discharging the different liquids for each step. After cleaning, tooth enamel samples were leached to remove and minimize diagenetic contamination from the enamel. Samples were left in 0.25 M acetic acid (CH_3_COOH) in an ultrasonic agitation bath for 10 min and let stand for ~ 6 h. Samples were transferred from centrifuge vials to Teflon™ beakers for dissolution. Teflon™ beakers were previously cleaned with 14 M HNO_3_ and rinsed with Milli-Q® water. One ml of 14 M HNO_3_ was added to each sample in the Teflon™ beakers. The Teflon™ beakers were closed and placed on a hotplate overnight at 150 °C. Once the samples were fully dissolved, the Teflon™ beakers were left open on the hotplate at 150 °C to let them evaporate.

#### Vegetation

Dried grass samples were cut into small pieces and transferred into cleaned Teflon™ beakers. Samples were directly dissolved in 10–15 ml of 14 M HNO_3_. Teflon™ beakers were placed on the hotplate at 150 °C overnight, then opened and left on a hotplate at 150 °C until full evaporation. These steps were repeated until the complete dissolution of samples (3–4 times).

#### Soil

Soil samples were moved into cleaned Teflon™ beakers and leached, adding 8 ml of 0.25 M acetic acid (CH_3_COOH) and left to stand for ~ 36 h. The leachate was extracted with a pipette and then centrifuged. The centrifuged liquid was transferred into pre-cleaned 15 ml Teflon™ beakers and dried down on a hotplate at 150 °C. To destroy any organic material left, 2 ml 14 M HNO_3_ was added into Teflon™ beakers and left overnight on the hotplate at 150 °C. The Teflon™ beakers were opened and placed on the hotplate at 150 °C to be fully evaporated.

#### Water

Water samples were dried down on the hotplate at 110 °C. The leftover residue was further treated with 0.5 ml 14 M HNO_3_ and placed on the hotplate at 150 °C for ~ 1 h. 14 M HNO_3_ was subsequently evaporated.

#### Snail shells

Snail shell samples were thoroughly cleaned with a DREMEL® dental bur. Samples were then dissolved in 2 ml of 14N HNO_3_ and then evaporated.

Sr from all samples was extracted using miniaturized extraction chromatographic columns filled with EICHROM Sr Spec Resin for separating Rb and Sr and removing the major matrix elements (such as Ca, P etc.). 300 μl of 3N HNO_3_ was added into Teflon™ beakers to re-dissolve the samples. Labelled Teflon™ beakers were placed on a hotplate at 100 °C for ~ 30–60 min. Samples were pipetted into assigned Teflon™ columns filled with 100 µl EICHROM Sr Spec Resin using acid-cleaned pipette tips. Labelled Rb Teflon™ beakers were placed under labelled columns. 950 μl of 3 M HNO_3_ was added into each Teflon™ column for the cleaning procedure. The discharged liquid was collected as Rb beakers, followed by 1200 μl of Milli-Q® water which was collected as Sr fraction. The Sr Teflon™ beakers were closed and placed on the hotplate at 100 °C for ~ 2 h to evaporate the samples fully. This separation step for the Sr fractions was repeated twice in total.

^87^Sr/^86^Sr ratios were measured in static mode using Neptune™ Multicollector ICPMS technology at the Frankfurt Isotope and Element Research Center (FIERCE) of the Goethe University (see Müller & Anczkiewicz^61^). The reproducibility of the Sr isotopic standard SRM987 during the course of analysis is given in Supplementary Tables S6 and S7 captions, and was always within error of the accepted value of 0.710245. Sr blank measurements were conducted using a dilute ^84^Sr-enriched tracer solution (“spike”) and found to be negligible relative to the relatively large Sr samples processed.

### FTIR (Fourier Transform Infrared Spectroscopy) analysis

Within a more extensive ^14^C study of the necropolises, FTIR analysis on 5 individuals (Supplementary Table S7) was performed at the ^14^CHRONO Centre for Climate, the Environment, and Chronology in the School of Natural and Built Environment at Queen’s University Belfast to check on the degree of cremation (crystallinity index or splitting factor).

To prepare a disc for FTIR, 1–1.5 mg of sample was mixed with 0.15–0.2 g of spectrosol grade KBr. This was ground using an agate pestle and mortar until the sample was less than 63 um. The sample was pressed into a pellet using a Specac hydraulic press, at 10,000 kg for 3 min. The disc was then analysed using a Perkin Elmer Spectrum One FTIR spectrometer. Samples with crystallinity above 5 were considered sufficiently re-crystallized^62^ and radiocarbon-dated. Samples were stored in a sealed vial before hydrolysis with stock (85%) orthophosphoric acid (15 ml per g of bone).

### Statistical analyses

The open-access software R (version 4.2) was used for statistical analyses and to plot data in graphs (quantile–quantile plots, kernel density distribution estimate plots, box–and–whiskers plots)^63^. The “assignR” package^37^ was used to test the provenance probability of the non-local individuals across the Italian isoscape^24^ (the R code is reported in the Supplementary Note). In brief, the method of Ma et al. uses a Bayesian approach, where the prior probability assumes that all grid cells are equally likely locations of origin of a specific individual. The posterior probability of sample origin is computed at each grid cell (based on the isotope ratio of the samples), returning a raster object which contains one probability density surface per sample with its likely provenance. Multiple samples can be combined together (joint probability) assuming they come from the same area. A variable prediction error (standard deviation), resulting from the Kriging interpolation, was associated to the isoscape cells. The used raster files are reported as examples in the supplement (Supplementary Fig. S8) and can be downloaded from: https://www.geochem.unimore.it/sr-isoscape-of-italy/.

## Supplementary Information

Supplementary Information 1.

## Data Availability

All the data are fully available within the Supplementary Information.
