# Supplementary material for: Intense community dynamics in the pre-Roman frontier site of Fermo (ninth–fifth century BCE, Marche, central Italy) inferred from isotopic data
Source: Sci Rep. 2023 Mar 3;13:3632. doi: 10.1038/s41598-023-29466-3 (PMC9984403; doi:10.1038/s41598-023-29466-3)
Supplement: Supplementary file 1 — Supplementary Information 1. [file 41598_2023_29466_MOESM1_ESM.docx]

**Intense community dynamics in the pre-Roman frontier site of Fermo (ninth-fifth century BCE, Marche, central Italy) inferred from isotopic data**

Carmen Esposito^1,2*^, Melania Gigante^3^, Federico Lugli^4,5^, Pasquale Miranda^6^, Claudio Cavazzuti^7^, Alessandra Sperduti^8,9^, Marco Pacciarelli^6^, Simon Stoddart^10^, Paula Reimer^2^, Caroline Malone^2^, Luca Bondioli^3,4^, Wolfgang Müller^11,12^

^1^ School of History, Archaeology and Religion, Cardiff University, Cardiff CF10 3EU, UK

2 School of Natural and Built Environment, Queen’s University Belfast, Belfast BT7 1NN, UK

^3^ Department of Cultural Heritage, University of Padua, Padua 35139, Italy

^4^ Laboratory of Osteoarchaeology and Paleoanthropology (Bones Lab), Department of Cultural Heritage, University of Bologna, Ravenna 48100, Italy

^5^ Department of Chemical and Geological Sciences, University of Modena and Reggio Emilia, Modena 41125, Italy

^6^ Department of Humanistic Studies, University of Naples Federico II, Naples 80133, Italy

^7^ Department of History Cultures Civilizations, University of Bologna, Bologna 40124, Italy

^8^ Bioarchaeology Service, Museum of Civilizations, Rome 00144, Italy

^9^ Department of Asian, African and Mediterranean Studies, University of Naples “L’Orientale”, Naples 80134, Italy

^10^ Department of Archaeology, University of Cambridge, Cambridge CB2 3ER, UK

^11^ Institute of Geosciences, Goethe University Frankfurt, Frankfurt am Main 60438, Germany

^12^ Frankfurt Isotope and Element Research Center (FIERCE), Goethe University Frankfurt, Frankfurt am Main 60438, Germany

*corresponding author

email: [cesposito01@qub.ac.uk](mailto:espositoc@cardiff.ac.uk)

# **Supplementary note**

**The Villanovan material culture, its origin and scientific debate.** The necropolis of Villanova di Castenaso near Bologna (Emilia-Romagna region, Italy), discovered in the mid-19^th^ century, gave the name to an Early Iron Age culture subsequently recognized in other areas of the peninsula^1^. Ever since, archaeologists have identified a number of specific criteria to classify a site as Villanovan. Firstly, the use of cremation is recognized as a distinctive and shared funerary ritual within the Villanovan necropolises. Cremated human remains are deposited in typical vases with a distinctive neck covered by in-turned rim bowls, which were used as lids to seal the urns. Both the urns and the bowls display diagnostic shapes and decorations. The cinerary urn is generally placed in a pit excavated in the earth and filled with “*terra di rogo*” – the pyre remnants (Supplementary Fig. S1). Nonetheless, these similarities in the funerary ritual are more consistent and remarkable in the earliest phases of the necropolises^2^, namely from the end of the 10^th^ to the 9^th^ century BCE. Finally, the settlement structure is also distinctive. Naturally defined sites in the form of plateaux or clustered hills located in strategic areas for the control of resources were occupied at the beginning of the Early Iron Age (EIA: end of the 10^th^–9^th^ century BCE), replacing smaller settlements typical of the Final Bronze Age (FBA: mid of the 12^th^–10^th^ century BCE). Some scholars have classified those settlements as proto-urban centres^3,4^, alternatively better known as forms of early state formation. The notion that the Villanovan material culture identifies a homogenous phenomenon representative of an ancient people is reinforced by the geographical and cultural continuity between the Villanovan and Etruscan groups, where the latter have been recognized as an ethnic group^5^.

Villanovan material culture is documented in modern Tuscany (northern Etruria), western Umbria (Perugia and its territory) and north Latium (southern Etruria) on the Tyrrhenian coast, a vast territory recognized as ancient Etruria^6^. Besides the Etruscan territory, four areas in the Italian Peninsula display Villanovan features (Fig. 1). These areas are located in: (a) the Campania region, focused on the main sites of Pontecagnano, Sala Consilina and Capua^7^; (b) the Emilia region, focused on the main site of Bologna and a territorial system of villages between the Idice and the Reno valleys, later on including the areas of Panaro and Santerno^8^; (c) the Romagna region, focused on the main site of Verucchio and a territorial system of villages in an area that extended from San Marino to Cesena^9^; and (d) the Marche region, centred on the site of Fermo.

Although similarities between the Villanovan sites of Etruria have reinforced the idea of a homogenous phenomenon, a more in-depth analysis points to some differences among Villanovan sites. One illustrative example is the ritual of cremation, which is generally considered an essential element for labelling a site as Villanovan (see above). Nonetheless, inhumations are widespread since the beginning in the necropolises of Capua and Pontecagnano. Cerveteri^10^ and Populonia^11^ – two main Villanovan sites that became Etruscan in historical times – record cremations and inhumations from their very first phases. By contrast, cremations were still widespread at Chiusi in the 6^th^ century BCE. Those similarities are even less consistent in the sites located outside Etruria, which display Villanovan features while developing a hybrid material culture interwoven with local practices. For this reason, archaeologists have for a long time debated the nature of Villanovan sites outside Etruria, mainly focusing on interpreting their ethnic identity and origin^12,13^. The issue of the Villanovan material culture can therefore be considered one of the most fundamental issues of Italian prehistory.

The multifaceted nature of the Villanovan sites outside Etruria has led archaeologists to propose a range of contrasting interpretations. One view held by traditional Etruscologists interprets the material culture as representative of a clear and distinct cultural group directly related to the Etruscans^1,14^. Consequently, the areas outside Etruria were identified as the outcomes of Villanovan/Proto-Etruscan expansion within the broader Italian territory. By contrast, Peroni, the leading Italian protohistorian of the second half of the last century, rejected the hypothesis proposed by Etruscologists and other scholars, considering it too simplistic an association between material culture and ethnic groups. He reasoned that the Villanovan material culture represented similar traits (e.g. funerary ritual, material culture and settlement structure) shared by groups of diverse origins. He proposed that these groups intentionally broke with traditional customs of the past and adopted new, shared settlement organizations and socio-political structures. Hence, Peroni interpreted Villanovan practice as a re-elaboration of an ideal prototype rather than the exchange of goods and movement of people^12^. The inconsistency of shared features in the material culture among the Villanovan sites within Etruria and, even more, the examples located outside Etruria further supported in his opinion this hypothesis. In Peroni’s view, those groups recognized themselves as ethnically bounded only in historical times through an Etruscan identity^12^, postponing the concept of the ethnic formation of the Etruscans until the 7^th^ century BCE.

**δ^13^C and δ^15^N isotopes.** Carbon (δ^13^C) and nitrogen (δ^15^N) stable isotope analyses performed on inhumed human remains provide information on long-term past feeding behaviours^15,16^. These analyses show foodstuff values incorporated in both human bone collagen, which mainly indicates protein intake, and bone apatite, reflecting more broadly proteins, carbohydrates and lipids^17–19^. By contrast, since the organic component does not resist high temperatures, these analyses of cremated remains can only provide data on the temperature and ventilation of the pyre in cremations^20^.

δ^13^C and δ^15^N in inhumed human remains can provide information on long-term diet. The estimation of δ^13^C in bone collagen discriminates the type of plant consumed by a specific population. For example, C3 plants (e.g. wheat, barley, oats, rice, potato, manioc and yam) – typical of cold weather regions – have a δ^13^C value range between -36 ‰ and -24 ‰^21^. Several factors, such as temperature^22^, water availability, light intensity and nutrient accessibility^16^, can influence these values. C4 plants (e.g. maize, sorghum, millets, sugarcane and tropical pasture grasses) – typical of tropical and dry habitats – have δ^13^C values around -16 ‰ to -8 ‰^22^. The δ^13^C values of C4 plants are less affected than C3 plants by environmental factors^23^. CAM plants (e.g. cactus, sedum, pineapple) – typical of hot and dry environments – have δ^13^C with intermediate values between C3 and C4 plants, namely between -20 ‰ to -10 ‰^22^. Environmental conditions such as salinity, day length, night temperature and water stress strongly influence δ^13^C values in CAM plants^16^.

δ^15^N values can identify carnivores against herbivores in the food chain. Since δ^15^N visibly increases along with the trophic level, primary consumers can be distinguished from secondary and tertiary consumers^24–26^. The increase of δ^15^N is +2–6 ‰ from diet to body protein, and this shift continues along the food chain^27,28^. An enrichment of 0 ‰ is expected in a vegan diet^29^. Based on the same principle, stable nitrogen isotope can identify weaning practices, with a δ^15^N enrichment of 2–4 ‰ occurring between breastfeeding infants and their mothers^30^. By contrast, the δ^13^C value shows only a small increase of 1–2 ‰ in collagen^21^ compared to that of the diet, but the reasons for this enrichment are not entirely explored^16^. Even if δ^15^N values in collagen mainly reflect protein intake, meat was likely to be a supplement to staple foods. Consequently, assuming that cereals and legumes made a significant contribution to past diet, δ^15^N in collagen often reflects values pertaining to that food. Low collagen δ^15^N values express cereal and legume consumption compared to more carnivorous diets^31^. Besides diet, environmental and cultural factors can also influence δ^15^N values^32,33^.

Combining δ^13^C and δ^15^N isotope values can give information on the consumption of marine food. δ^13^C values in marine plants and phytoplankton range around -19.5 ‰^22^. Likewise, δ^15^N values in fish and mammals are higher than in terrestrial fauna^34^ due to a more significant amount of bacterial denitrification. Additionally, higher δ^15^N values are also influenced by the stepwise enrichment in δ^15^N in long marine food chains^23^. Consequently, δ^15^N values in bone collagen of humans with marine-based diets are around +17 ‰ to +20 ‰, while humans who fed on plants and terrestrial animals have lower values, around +6 ‰ to +12 ‰^34^. It is important to stress that spotting possible marine food consumption in human bones can support the interpretation of other isotope analyses such as strontium (^87^Sr/^86^Sr). The consumption of fish can indeed largely influence the ^87^Sr/^86^Sr isotope values in human individuals, which will be closer to the seawater ^87^Sr/^86^Sr values (0.7092 by definition). Therefore, identifying a diet based on marine food can benefit ^87^Sr/^86^Sr analysis.

Bone goes through a continuous turnover, which varies according to the type of bone sampled. Collagen is synthesized at various stages of an individual’s lifespan. For this reason, many studies have compared different types of bone in the same individual (e.g. ribs vs femurs) to spot variability in the diet. Femurs reflect a longer-term diet, namely far above 10 years before death^28^, while ribs recall diet from the last years of a person’s life^35^.

**^87^Sr/^86^Sr isotope.** The strontium isotopic ratio signature (^87^Sr/^86^Sr) of a certain geological area depends on (a) the original composition of the rock, i.e. the original Rb–Sr ratio at the time the rock crystallized, and (b) the radioactive decay of long-lived radionuclides (^87^Rb) to stable daughter isotopes of a diverse element (^87^Sr)^36,37^. Hence, the isotopic signatures in different geological formations can approximately vary as follows: (a) pure carbonates <0.710 (0.706–0.709); (b) granites, gneisses (>0.710); and (c) basalts <0.706^38^. The ^87^Sr/^86^Sr isotopic ratio passes from geological environment (i.e. local bedrock geology) to soil and groundwater through weathering processes, and from these latter to local plants and animals^39,40^. It eventually reaches, almost unvaried, human mineralized hard tissues (i.e. tooth enamel and bone) through diet.

Tooth enamel and the otic capsule of the temporal bone (pars petrosa) are sampled and compared to local ^87^Sr/^86^Sr outcomes – generally identifiable at the place of burial – to spot possible non-local individuals at a site. Indeed, the pars petrosa and most permanent tooth enamel start forming in utero and in early childhood^41^ respectively and do not change once formed. If ^87^Sr/^86^Sr values in the human samples differ from the local ^87^Sr/^86^Sr values, the analysed individual might have spent his/her childhood elsewhere. Alternatively, this difference could indicate that the individual consumed food and water from an area other than the one around the cemetery.

The most targeted teeth in inhumation are the permanent first molar (M1) or permanent second molar (M2) tooth enamel and, more rarely, the third molar (M3). The first lower M1 crown generally starts to form before birth, as witnessed by the presence of the neonatal line, an accentuated incremental line forming at birth^42^ until 3 years of age. The M2 tooth crown begins to form at 3 to 4 until 7 to 8 years of age. M3 formation and eruption vary significantly among individuals and can start between 6 and 12^43,44^ until 24 years of age.

The most targeted sample for cremations is the pars petrosa^45^, since tooth crowns are rarely found in cremated human remains due to heat effects, with some exceptions (i.e. developing teeth that are protected inside the mandible or the maxilla^46^). This is possible because crystallized bone tissues resulting from high temperatures preserve *in vivo* strontium and are far more resistant to the process of diagenesis^47^ compared to unburned bones.

**The challenge of assessing the local ^87^Sr/^86^Sr baseline.** Assessing the local ^87^Sr/^86^Sr ratio is a fundamental step to intercepting non-local individuals among the analysed samples. In pioneer studies, the local ^87^Sr/^86^Sr values were measured on the local geology^48^. However, recent work has demonstrated that those values only partially reflect human ^87^Sr/^86^Sr ratio values. Complex weathering of the bedrock^49,50^ and atmospheric inputs^36,51^ can largely influence local bedrock ^87^Sr/^86^Sr values. In contrast to the variability observed for environmental samples, humans’ tissues generally show more homogenous ^87^Sr/^86^Sr values. Humans indeed consume a wider variety of foodstuffs, which is averaged^52^. For this reason, two-component or three-component mixing models^50^ which take into consideration drinking water, vegetation and fauna to predict the local ^87^Sr/^86^Sr range of an archaeological site have been proposed. However, these methods seem to underestimate the locally bioavailable ^87^Sr/^86^Sr ratios when the sample size is too limited. For this reason, a combined approach that takes into account diverse methodologies to identify ^87^Sr/^86^Sr local signatures is so far considered a suitable method to reach reliable local ^87^Sr/^86^Sr values^50,53,54^.

**The local biologically available strontium (BASr).** Diverse studies have pointed out the validity of local biologically available strontium (BASr) to construct a solid local baseline. This methodology, indeed, considers the various ^87^Sr/^86^Sr sources which enter the system and contribute, together with the bedrock ^87^Sr/^86^Sr values, to the ^87^Sr/^86^Sr values found in plants, water, soil and fauna^51,55^. The most suitable approach is to collect a variety of samples such as plants, water, soil and archaeological and modern fauna for constructing a local baseline. Indeed, all those samples display advantages and disadvantages due to their intrinsic archaeological and environmental biases^40^.

**The shape of the sample distribution of the human data.** Some studies have proposed statistical methods to identify possible outliers (i.e. non-local individuals) in the analysed population, spotting values that do not fit the distribution of the majority of data^56^. Providing the sample number is consistent enough (e.g. >25 see, for oxygen analyses^57^), statistical approaches show the advantage of avoiding comparison between different types of samples (e.g. human samples versus plants, fauna, soil). Wright^56^ and more recently Sengeløv *et al*.^37^ have assumed that ^87^Sr/^86^Sr human values of a population – where most individuals are expected to be local – are normally distributed. In both studies, human samples were plotted along with the normal distribution, namely Gaussian. However, if this approach is adopted, some considerations must be taken into account. Firstly, this methodology cannot be applied to mass migrations, major battlegrounds and extremely mobile populations^58^ since, in these cases, the majority of individuals are non-local. Secondly, the site under examination should not have substantial variability in the local geology. Finally, sites characterized by a significant import of food from distant areas must be excluded.

**Baseline on subadults.** A few studies^54,59–61^ have proved the validity of employing ^87^Sr/^86^Sr values in subadult individuals to detect the local baseline. Children aged 1 to 10 years are the most relevant for this purpose. The underlying principle is that permanent tooth enamel in subadults forms shortly before death, and, consequently, a limited possibility of residential mobility can be inferred^54^. Kernel density plots – which display the ^87^Sr/^86^Sr distribution of adult and subadult individuals over a continuous interval – demonstrate in various studies^50,61^ that subadults’ ^87^Sr/^86^Sr values are distributed where the majority of adult individuals’ values are. Furthermore, their ^87^Sr/^86^Sr values correspond to the local baseline^61^. Nonetheless, this approach also needs some consideration for possible biases. Firstly, children show slightly more restricted ^87^Sr/^86^Sr values than adults, which might be due to a less varied diet. Additionally, the residential mobility of children cannot be excluded^62,63^. Finally, it is not always easy to find subadults’ burials within necropolises due to cultural reasons. Consequently, it seems preferable to choose more than one type of method when assessing local ^87^Sr/^86^Sr since all the approaches mentioned above can display potential biases.

**Rcode for the provenance estimate**

**##################################################################################**

**########################################################################################## R code for provenance assignment of Esposito et al. 2023 Sci. Rep. #######**

**####### The code is based on the Ma et al. 2020 paper #############################**

**####### Ma, C., Vander Zanden, H. B., Wunder, M. B., & Bowen, G. J. (2020).#######**

**####### assignR: An R package for isotope-based geographic assignment.############**

**####### Methods in Ecology and Evolution, 11(8), 996-1001. ########################**

**#############################################################################################################**

**############################################################################################################**

**vignette('assignR') #Carefully read the assignR vignette before starting**

**##########################################################################################################**

**####Required packages**

**#library(raster)**

**#library(assignR)**

**#library(rgdal)**

**#library(tidyverse)**

**####Set directory of raster isoscape maps**

**setwd("~/R_data")**

**####Load the needed raster maps**

**#for Italy, you can download them from https://www.geochem.unimore.it/sr-isoscape-of-italy/**

**sr<-raster("UK_bioav_ANNA.tif") #Sr isoscape raster**

**crs(sr) <-**

**"+proj=longlat +datum=WGS84 +no_defs +ellps=WGS84 +towgs84=0,0,0" #set the proper coordinate reference system**

**sr.err<-raster("UK_bioav_ANNA_error.tif") #prediction error raster**

**sr.err<-sqrt(sr.err) #converting the raster as standard deviation, the original raster is reported as variance**

**crs(sr.err) <-**

**"+proj=longlat +datum=WGS84 +no_defs +ellps=WGS84 +towgs84=0,0,0" #set the proper CRS**

**sr.stack<-stack(sr, sr.err) #stack the two raster in the same object**

**plot(sr.stack) #check whether the maps plot properly**

**####Generate a data frame with the 87Sr/86Sr data of your individuals**

**id=c("X59D","X59A","X78","X17") #samples' names**

**sr.dat = c(0.709678, 0.709528, 0.709517, 0.709581) #isotope ratios**

**sr.sd = c(0.000017, 0.000017, 0.000022, 0.000022) #associated standard errors**

**unk.sr <- data.frame(id, sr.dat, sr.sd)**

**####Probability assignment for each individual**

**unk.sr.prob <- pdRaster(sr.stack, unknown=unk.sr)**

**plot(unk.sr.prob) #check the individuals' provenances**

**####Joint probability for all the samples together**

**#from the vignette: "jointP calculates the probability that all**

**#samples came from each grid cell**

**#in the analysis area. Note that this summarization will only**

**#be useful if all samples are truly**

**#derived from a single population of common geographic**

**#origin."**

**joint.sr<-jointP(unk.sr.prob)**

**####Best 10% assignment**

**#from the vignette: "10% of the study area, giving maps that**

**#show the 10% of grid cells with the**

**#highest posterior probability for each sample."**

**top_prob<-qtlRaster(joint.sr, threshold = 0.1)**

**#######Distance and direction**

**#######INDIVIDUALLY**

**####give to each individual the coordinates of finding**

**#if the individuals comes from the same site (as in this case)**

**#just insert the coordinates of the site for all of them**

**pp1 = c(13.715, 43.170)**

**pp2 = c(13.715, 43.170)**

**pp3 = c(13.715, 43.170)**

**pp4 = c(13.715, 43.170)**

**pp.all = SpatialPoints(coords = rbind(pp1,pp2,pp3,pp4)) #bind all the coordinates together in a single object**

**proj4string(pp.all) = proj4string(sr) #assign proper CRS**

**####check the individual assignment map and the individual place**

**#of origin on the map**

**ID<-1 #by changing the ID number (i.e. individual order) you can run the plot**

**#and check the individual provenance + the location of origin site**

**plot(unk.sr.prob[[ID]], main = names(unk.sr.prob)[ID])**

**points(pp.all[ID])**

**####Run the probability of direction and provenance for each individual**

**wd = wDist(unk.sr.prob, pp.all[1:4])**

**####plot the graphs for direction of movement for each individual**

**c(wd)[c(1,2,4,6,8,10,12,14,16)]**

**plot(wd)**

**####Plot prob density of distance by ggplot**

**ID2<-"X17" #choose the individual of interest**

**wd.dens<-data.frame(wd[[ID2]][["d.dens"]][["x"]], wd[[ID2]][["d.dens"]][["y"]])#extract the data**

**names(wd.dens)<-c("distance","prob.density")#rename variables**

**ggplot(wd.dens)+ #plot**

**geom_line(aes(x=distance, y=prob.density))+**

**xlab("Distance (m)")+**

**ylab("Probability density")**

**########JOINT**

**####plot the joint probability + the place of origin of the individuals**

**plot(joint.sr, main = names(joint.sr))**

**points(pp.all[1]) #being the same for all the individuals,**

**#you can select one of them as the origin**

**####joint direction of movement**

**wd.join = wDist(joint.sr, pp.all[1])**

**c(wd.join)[c(1,2,4,6,8,10,12,14,16)]**

**plot(wd.join)**

**####Plot the joint probability density of distance by ggplot**

**wd.dens.joint<-data.frame(wd.join[["Joint_Probability"]][["d.dens"]][["x"]], wd.join[["Joint_Probability"]][["d.dens"]][["y"]])#extract the data**

**names(wd.dens.joint)<-c("distance","prob.density") #rename variables**

**ggplot(wd.dens.joint)+ #plot**

**geom_line(aes(x=distance, y=prob.density), colour="red", size=1)+**

**xlab("Distance (m)")+**

**ylab("Probability density")+**

**theme(panel.border = element_rect(fill=NA, colour = "black",**

**size=1))+**

**theme(panel.grid.major = element_blank(), panel.grid.minor = element_blank())+**

**theme(text = element_text(size = 16))**

# **Supplementary figures**

Figure S1. An exemplified figure of a Villanovan cremation. The urn covered by a bowl is located in a pit excavated in the earth together with “*terra di rogo*” – the pyre ashes – and then covered by a stone slab.

| 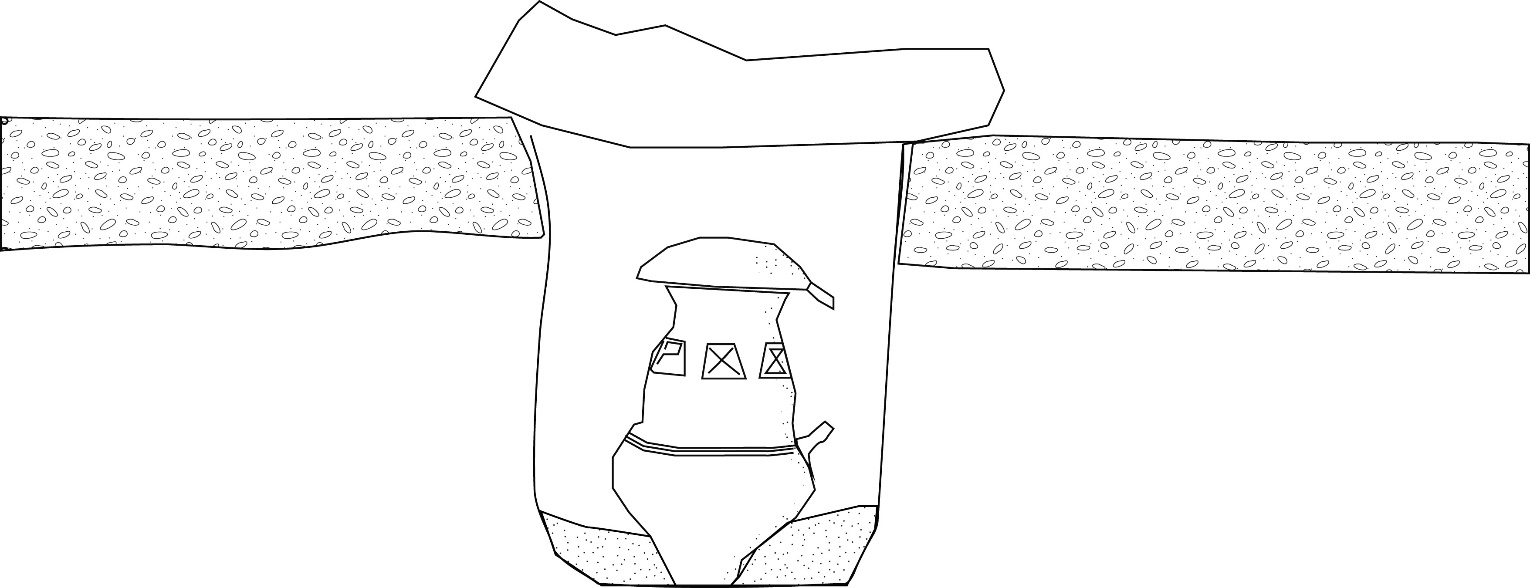 |
| --- |

Figure S2. The Misericordia and Mossa necropolises of Fermo (Marche, Italy). The Misericordia (red) and Mossa (blue) necropolises are located at the opposite sides of the Girfalco hill. Maps Data: Google, ©2022 CNES/Airbus, Maxar Technologies.


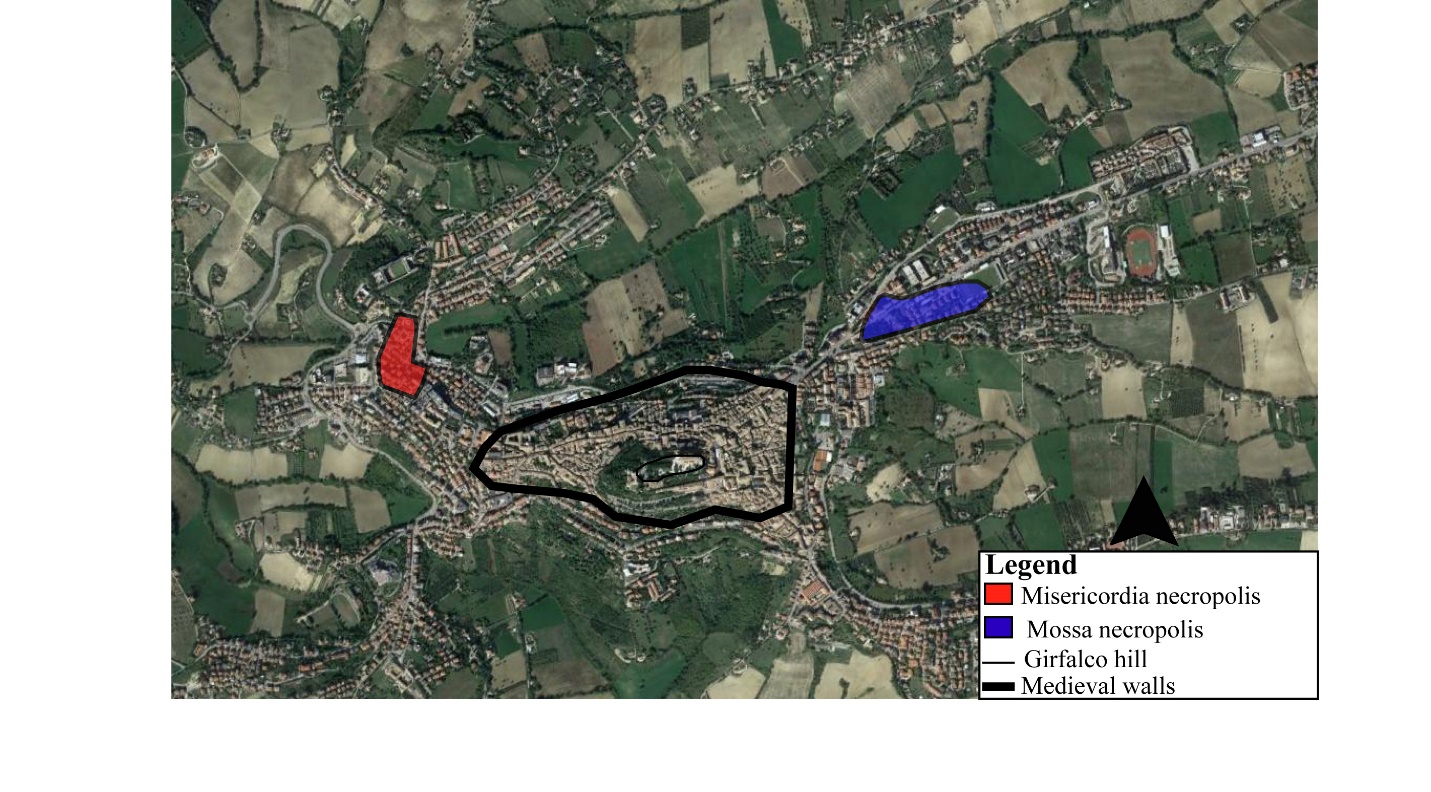


Figure S3. Geological map and isoscape of central Italy. (a) Geological map of Central Italy. This map is built in QGIS 3.18 (https://qgis.org), based on the geolithological map of Italy available at the Geoportale Nazionale. (b) Zoom of the Italian Sr isoscape to highlight the isotope distribution of the area of interest (see Lugli *et al*.^64^). Around Fermo (range of ca. 8–10 km), the isoscape shows values ranging between 0.7088 and 0.7090. The map is built using QGIS 3.18 (https://qgis.org) and SAGA 2.3.2 (https://saga-gis.sourceforge.io/).


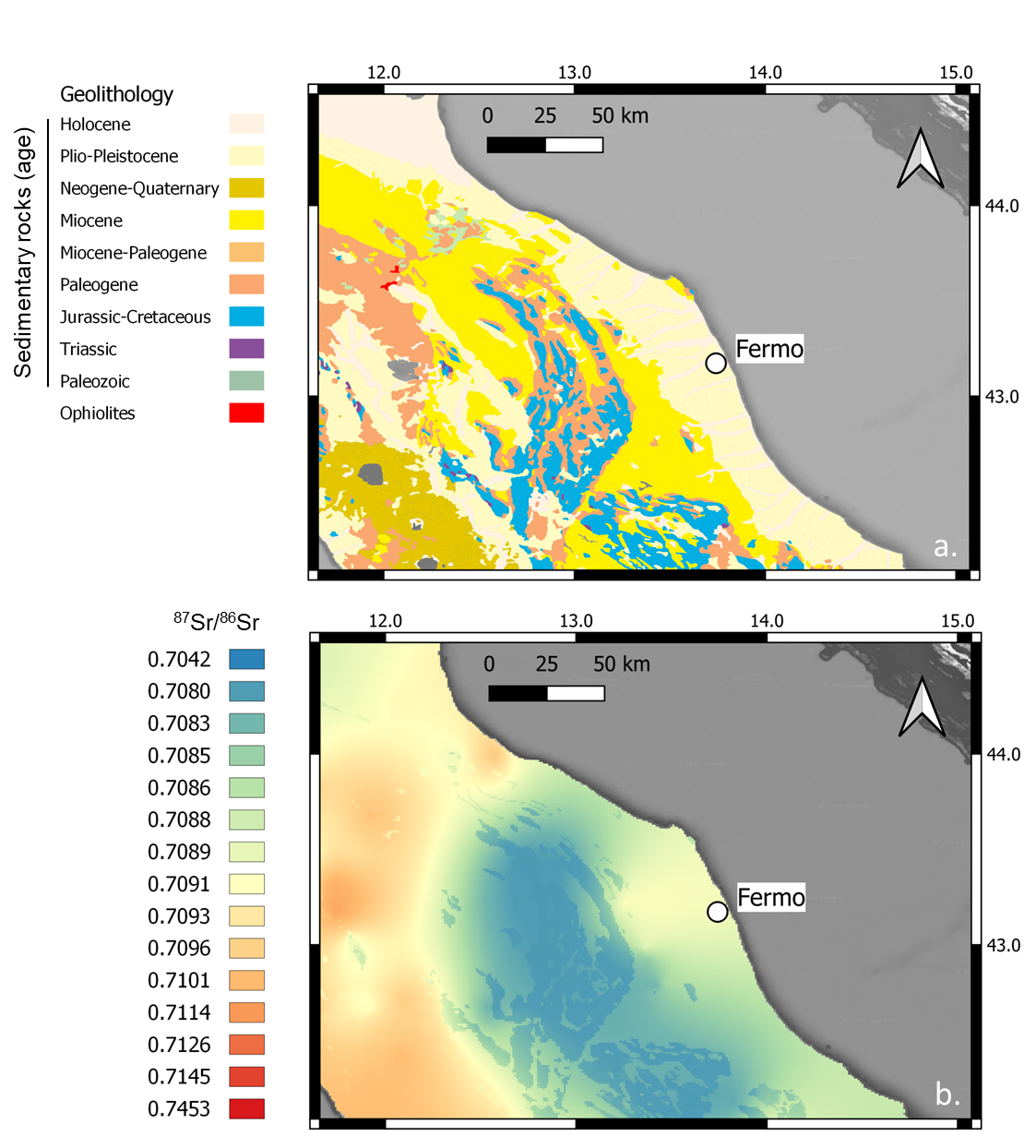


Figure S4. Box–and–whiskers plot showing ^87^Sr/^86^Sr values of the baseline and human samples analysed in this study. Baseline samples (n = 11) include snail shells, fauna tooth enamel, soil, water and vegetation collected at 0–7 km distance from Fermo.


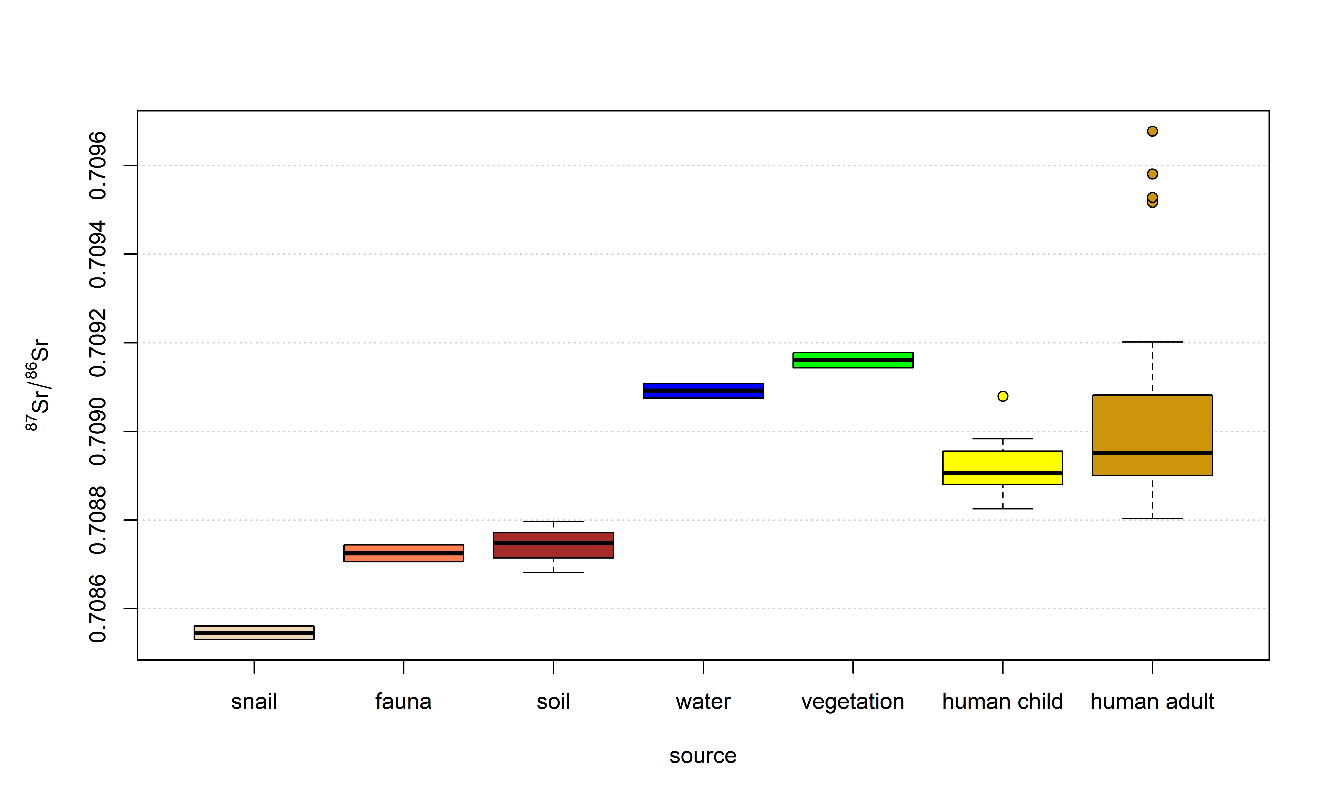


Figure S5. Normal probability plot (Q–Q plot) of ^87^Sr/^86^Sr values of Fermo human samples. The Q–Q plot compares the expected normal (red line) versus data quantiles. Red dots: females (F, F(?)); blue dots: males (M, M(?)); green dots: juveniles; orange dots: older children (5–10 years of age); yellow dots: young children (1–5 years of age); grey dots: adults indeterminate; black polygons indicate outlying values excluded from the local sample. At least, 9 out of 54 samples visibly stand out from the normal distribution. The central tendency of the human data at Fermo, which is given by our entire sample except for the 9 outliers identified in the Q–Q plot, is 0.70882 to 0.70909.


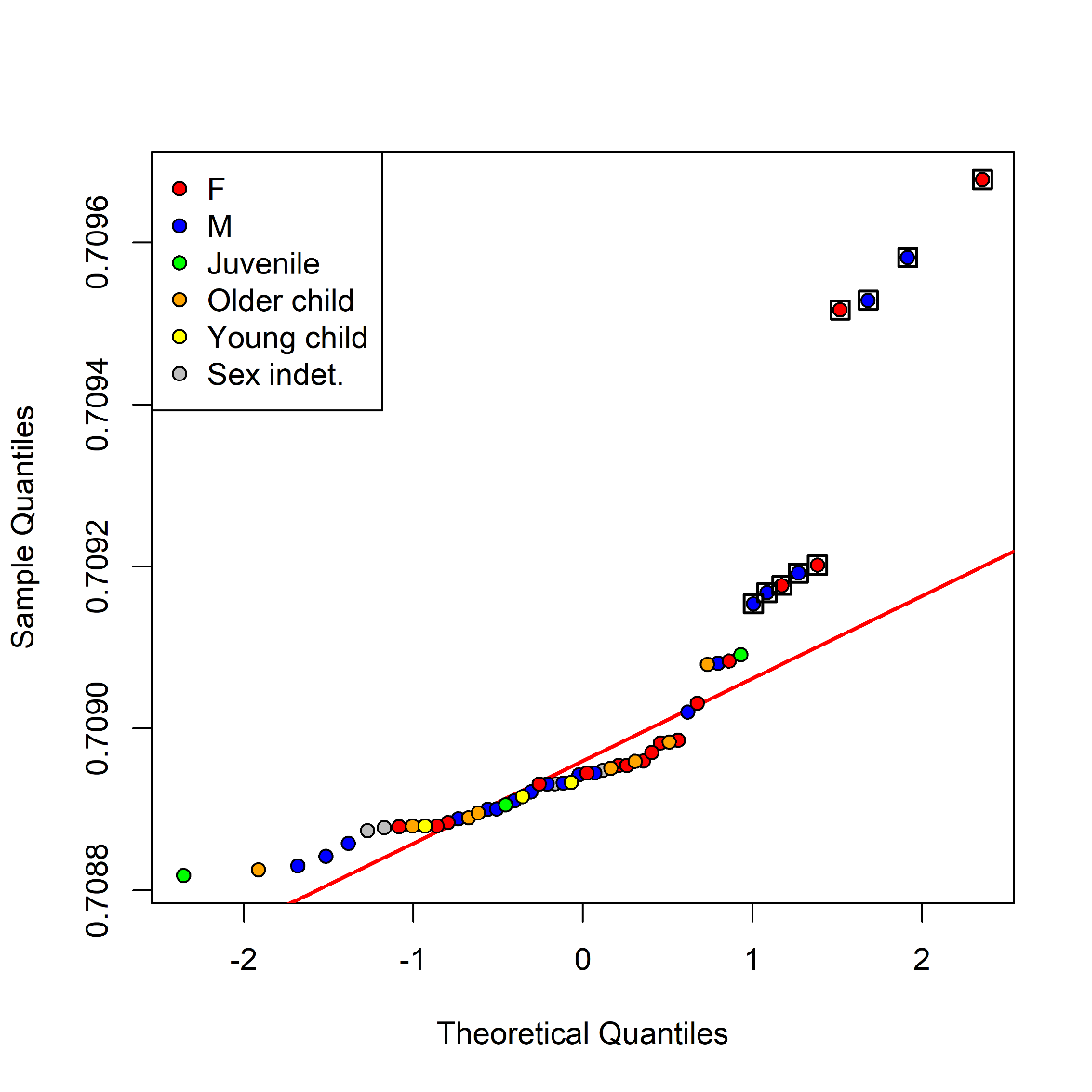


Figure S6. Density plot of human ^87^Sr/^86^Sr values for Fermo. Red dots: females (F, F(?)); blue dots: males (M, M(?)); green dots: juveniles; orange dots: older children (5–10 years of age); yellow dots: young children (1–5 years of age); grey dots: adults indeterminate. Yellow lines: young children (YC = 1–5 years of age) range; orange lines: older children (OC = 5–10 years of age) range. The purple dashed lines indicate the Tukey interquartile range method (1.5xIQR). A density plot visualizes data distribution over a continuous interval – the peaks of a density plot display where values are concentrated. A density plot is usually described as a “smoothed” histogram of the sample distribution. The type of the kernel and its size influences the strength of smoothing and should be carefully chosen to avoid the introduction of artefacts. For this work, the most commonly adopted Gaussian kernel that fits the expected base distribution and the default bandwidth used by the “stats” package of R0 was used. The red line is the density estimate of the ^87^Sr/^86^Sr values. A narrower ^87^Sr/^86^Sr range for young and older children, yellow and orange lines respectively (0.70883 to 0.70908), includes the higher peak in the kernel density estimate.


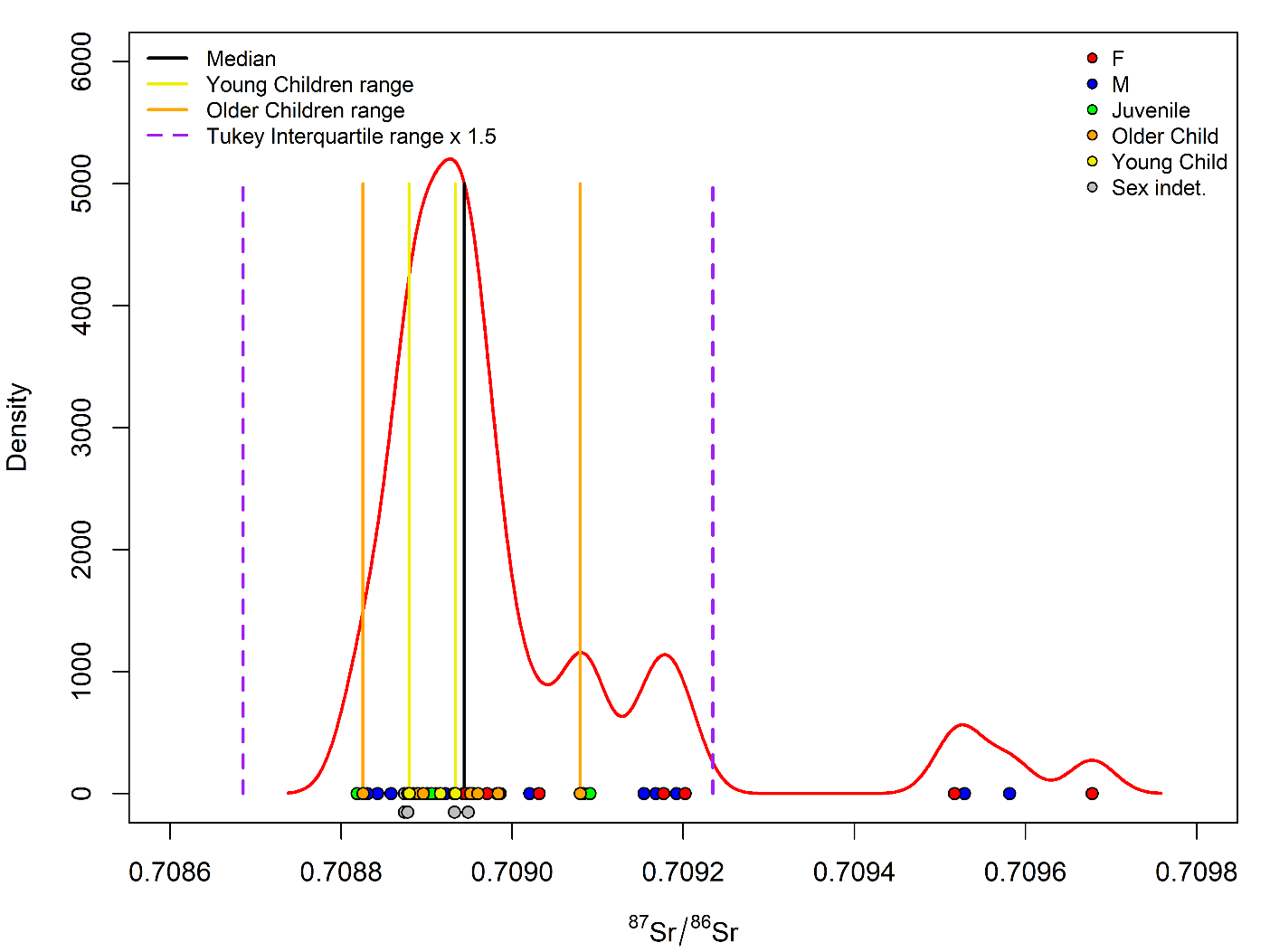


Figure S7. Tooth enamel dental sampling: (a) tooth in resin with dentine; (b) tooth in resin with dentine removed; (c) tooth without resin.


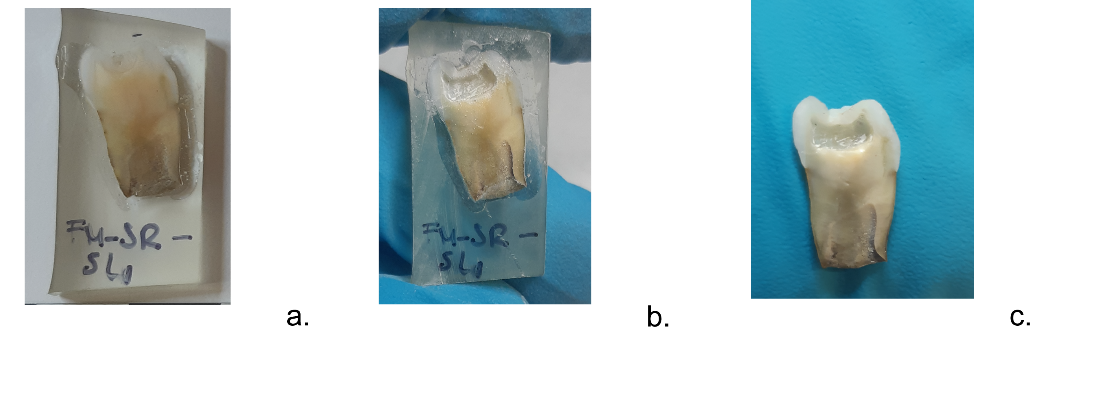


Fig. S8. (a) Universal Kriging model of the Sr isotope distribution in Italy, based on Lugli *et al*.^64^. (b) Prediction error of the Kriging model reported as standard deviation. Both maps are built using QGIS 3.18 (https://qgis.org) and SAGA 2.3.2 (https://saga-gis.sourceforge.io/).


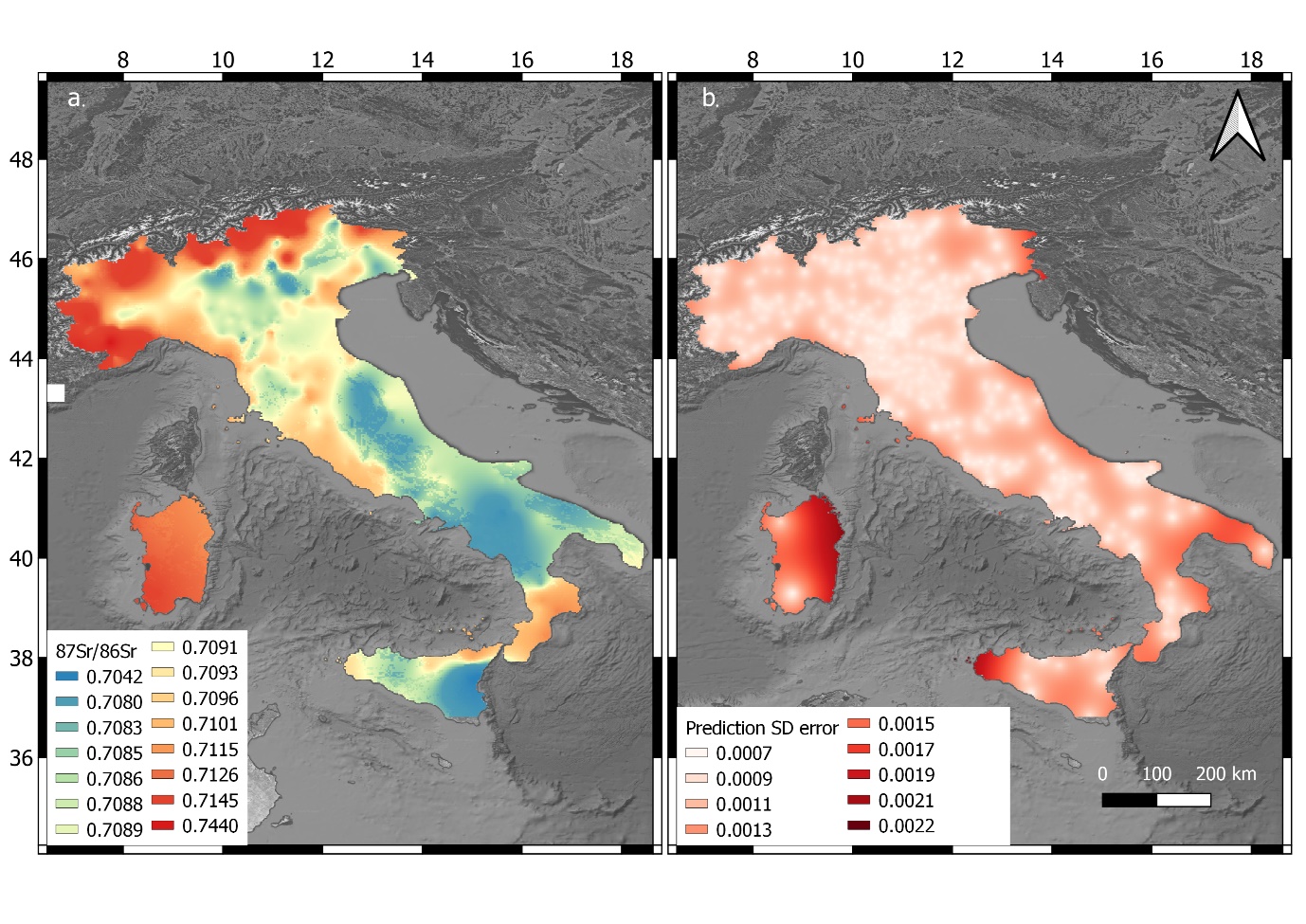


# **SUPPLEMENTARY TABLES**

Table S1. Tombs excavated at the Fermo Misericordia and Mossa necropolises, according to archaeological reports. The first sporadic archaeological finds at the Fermo Misericordia necropolis were brought to light in the 19^th^ century. Even though several archaeological campaigns occurred at the beginning of the 20^th^ century, excavations conducted with modern methodologies took place only later in the 1950s. In total 199 tombs were excavated in the Misericordia necropolis. The first scientific excavations took place at the Mossa necropolis in 1968, when 14 graves including both cremations and inhumations were found. Subsequently, in the 1980s and 1999–2000s, another 132 tombs were excavated. The excavation column indicates the year when the excavation took place, while the names in brackets are the landowners’ names. Overall, the grand total of 345 graves represents an estimate.

| **excavation** | **necropolis** | **n graves** |
| --- | --- | --- |
| 1908 excavation | MISERICORDIA | 3 |
| 1911 excavation | MISERICORDIA | 16 |
| Bonfigli 1956 | MISERICORDIA | 60 |
| Bonfigli 1959 (Alidori) | MISERICORDIA | 32 |
| Brusadin 1956–1957 | MISERICORDIA | 86 |
| Bonfigli (Sardellini) | MISERICORDIA | 2 |
| Lollini 1968 | MOSSA | 14 |
| Baldelli 1983–1984 | MOSSA | 30 |
| 1999–2000 excavation | MOSSA | 102 |
| Total: |  | 345 |

Table S2. Summary of the analysed tombs. Chron: A = end of the 9^th^ century to the beginning of the 8^th^ century BCE; A(?) = probable end of the 9^th^ century to the beginning of the 8^th^ century BCE; B = second half of the 8^th^ century BCE; C = 7^th^ and 6^th^ century BCE; IND = indeterminate. Ritual: C = cremation; I = inhumation; Sex: F = female; F(?) = probable female; M = male; M(?) = probable male; IND = indeterminate. Age: NI (newborn and early infant) = 0–1 years of age; YC (young child) = 1–5 years of age; OC (older child) = 5–10 years of age; J (juvenile) = 10–15 years of age; YA (young adult) = 16–20 years of age; MA (middle adult) = 20–40 years of age; OA (old adult) = 40+ years of age; GA (generic adult) = 20+ years of age. The assessment of the tomb archaeological chronology is based on various work^65–67^ and is still in progress.

| **n** | **excavation** | **necropolis** | **tomb**  **id** | **ind**  **id** | **burial typology** | **ritual** | **chron** | **sex** | **age**  **class** | **age**  **group** |
| --- | --- | --- | --- | --- | --- | --- | --- | --- | --- | --- |
| 1 | Fm-Mos-1968 | MOSSA | 2 | 1 | collective | I | C | M | 40+ | OA |
| 2 | Fm-Mos-1968 | MOSSA | 2 | 2 | collective | I | C | M | 40+ | OA |
| 3 | Fm-Mos-1968 | MOSSA | 2 | 3 | collective | I | C | M | 40+ | OA |
| 4 | Fm-Mos-1968 | MOSSA | 2 | 4 | collective | I | C | IND | 5–10 | OC |
| 5 | Fm-Mos-1968 | MOSSA | 6 | 1 | double | I | C | M | 40+ | OA |
| 6 | Fm-Mos-1968 | MOSSA | 6 | 2 | double | I | C | M | 40+ | OA |
| 7 | Fm-Mos-1968 | MOSSA | 11 | 1 | collective | I | B | IND | 20+ | GA |
| 8 | Fm-Mos-1968 | MOSSA | 11 | 2 | collective | I | B | F | 40+ | OA |
| 9 | Fm-Mos-1968 | MOSSA | 11 | 3 | collective | I | B | IND | 10–15 | J |
| 10 | Fm-Mos-1968 | MOSSA | 11 | 4 | collective | I | B | IND | 1–5 | YC |
| 11 | Fm-Mos-1968 | MOSSA | 11 | 5 | collective | C | B | F(?) | 20+ | GA |
| 12 | Fm-Mos-99-00 | MOSSA | 3 | 1 | collective | I | C | IND | 0–1 | NI |
| 13 | Fm-Mos-99-00 | MOSSA | 3 | 2 | collective | I | C | F | 20–40 | MA |
| 14 | Fm-Mos-99-00 | MOSSA | 3 | 3 | collective | I | C | F | 20–40 | MA |
| 15 | Fm-Mos-99-00 | MOSSA | 3 | 4 | collective | I | C | F | 40+ | OA |
| 16 | Fm-Mos-99-00 | MOSSA | 3 | 5 | collective | I | C | M | 40+ | OA |
| 17 | Fm-Mos-99-00 | MOSSA | 3 | 6 | collective | I | C | F | 15–20 | YA |
| 18 | Fm-Mos-99-00 | MOSSA | 3 | 7 | collective | I | C | F | 20–40 | MA |
| 19 | Fm-Mos-99-00 | MOSSA | 5 | 1 | single | I | B | M | 40+ | OA |
| 20 | Fm-Mos-99-00 | MOSSA | 7 | 1 | single | I | IND | M | 40+ | OA |
| 21 | Fm-Mos-99-00 | MOSSA | 10 | 1 | single | I | IND | F(?) | 20–40 | MA |
| 22 | Fm-Mos-99-00 | MOSSA | 17 | 1 | single | I | B | M | 40+ | OA |
| 23 | Fm-Mos-99-00 | MOSSA | 21 | 1 | single | C | B | F | 20+ | GA |
| 24 | Fm-Mos-99-00 | MOSSA | 29 | 1 | single | I | C | IND | 5–10 | OC |
| 25 | Fm-Mos-99-00 | MOSSA | 31 | 1 | single | C | B | F | 20–40 | MA |
| 26 | Fm-Mos-99-00 | MOSSA | 42 | 1 | single | I | IND | F | 20–40 | MA |
| 27 | Fm-Mos-99-00 | MOSSA | 44 | 1 | double | I | IND | IND | 15–20 | YA |
| 28 | Fm-Mos-99-00 | MOSSA | 44 | 2 | double | I | IND | IND | 5–10 | OC |
| 29 | Fm-Mos-99-00 | MOSSA | 51 | 1 | single | I | B | M | 20–40 | MA |
| 30 | Fm-Mos-99-00 | MOSSA | 56 | 1 | single | C | B | IND | 10–15 | J |
| 31 | Fm-Mos-99-00 | MOSSA | 58 | 1 | collective | I | B | IND | 40+ | OA |
| 32 | Fm-Mos-99-00 | MOSSA | 58 | 2 | collective | I | B | IND | 5–10 | OC |
| 33 | Fm-Mos-99-00 | MOSSA | 58 | 3 | collective | C | B | M | 40+ | OA |
| 34 | Fm-Mos-99-00 | MOSSA | 59D | 1 | double | C | B | F | 20+ | GA |
| 35 | Fm-Mos-99-00 | MOSSA | 59A | 1 | double | C | B | M | 20+ | GA |
| 36 | Fm-Mos-99-00 | MOSSA | 62 | 1 | double | I | B | IND | 5–10 | OC |
| 37 | Fm-Mos-99-00 | MOSSA | 62 | 2 | double | I | B | IND | 20–40 | MA |
| 38 | Fm-Mos-99-00 | MOSSA | 63 | 1 | single | C | B | M | 20+ | GA |
| 39 | Fm-Mos-99-00 | MOSSA | 64 | 1 | single | I | B | M | 40+ | OA |
| 40 | Fm-Mos-99-00 | MOSSA | 65 | 1 | double | I | B | IND | 10–15 | J |
| 41 | Fm-Mos-99-00 | MOSSA | 65 | 2 | double | I | B | IND | 5–10 | OC |
| 42 | Fm-Mos-99-00 | MOSSA | 67 | 1 | collective | I | C | M | 40+ | OA |
| 43 | Fm-Mos-99-00 | MOSSA | 67 | 2 | collective | I | B | IND | 40+ | OA |
| 44 | Fm-Mos-99-00 | MOSSA | 67 | 3 | collective | I | B | M | 40+ | OA |
| 45 | Fm-Mos-99-01 | MOSSA | 68 | 1 | double | I | C | M | 40+ | OA |
| 46 | Fm-Mos-99-02 | MOSSA | 68 | 2 | double | I | C | F | 40+ | OA |
| 47 | Fm-Mos-99-00 | MOSSA | 70 | 1 | single | I | B | IND | 5–10 | OC |
| 48 | Fm-Mos-99-00 | MOSSA | 78 | 1 | single | C | B | F(?) | 20+ | GA |
| 49 | Fm-Mos-99-00 | MOSSA | 81 | 1 | collective | I | C | IND | 1–5 | YC |
| 50 | Fm-Mos-99-00 | MOSSA | 81 | 2 | collective | I | C | IND | 1–5 | YC |
| 51 | Fm-Mos-99-00 | MOSSA | 81 | 3 | collective | I | C | IND | 1–5 | YC |
| 52 | Fm-Mos-99-00 | MOSSA | 81 | 4 | collective | I | C | M | 20–40 | MA |
| 53 | Fm-Mos-99-00 | MOSSA | 81 | 5 | collective | I | C | IND | 5–10 | OC |
| 54 | Fm-Mos-99-00 | MOSSA | 81 | 6 | collective | I | C | M | 20–40 | MA |
| 55 | Fm-Mos-99-00 | MOSSA | 81 | 7 | collective | I | C | M | 40+ | OA |
| 56 | Fm-Mos-99-00 | MOSSA | 81 | 8 | collective | I | C | F | 40+ | OA |
| 57 | Fm-Mos-99-00 | MOSSA | 81 | 9 | collective | I | C | IND | 1–5 | YC |
| 58 | Fm-Mos-99-00 | MOSSA | 81 | 10 | collective | I | C | IND | 0–1 | NI |
| 59 | Fm-Mos-99-00 | MOSSA | 81 | 11 | collective | I | C | M | 20–40 | MA |
| 60 | Fm-Mos-99-00 | MOSSA | 81 | 12 | collective | I | C | F | 20–40 | MA |
| 61 | Fm-Mos-99-00 | MOSSA | 81 | 13 | collective | I | C | F | 40+ | OA |
| 62 | Fm-Mos-99-00 | MOSSA | 81 | 14 | collective | I | C | M | 40+ | OA |
| 63 | Fm-Mos-99-00 | MOSSA | 83 | 1 | single | C | B | M(?) | 20+ | GA |
| 64 | Fm-Mos-99-00 | MOSSA | 84 | 1 | single | C | B | M | 20+ | GA |
| 65 | Fm-Mos-99-00 | MOSSA | 85 | 1 | single | I | C | M(?) | 20+ | GA |
| 66 | Fm-Mos-99-01 | MOSSA | 87 | 1 | collective | I | C | IND | 15–20 | YA |
| 67 | Fm-Mos-99-02 | MOSSA | 87 | 2 | collective | I | C | F | 20–40 | MA |
| 68 | Fm-Mos-99-03 | MOSSA | 87 | 3 | collective | I | C | M | 20–40 | MA |
| 69 | Fm-Mos-99-00 | MOSSA | 88 | 1 | collective | I | C | M | 40+ | OA |
| 70 | Fm-Mos-99-00 | MOSSA | 88 | 2 | collective | I | C | IND | 40+ | OA |
| 71 | Fm-Mos-99-00 | MOSSA | 88 | 3 | collective | I | C | M(?) | 40+ | OA |
| 72 | Fm-Mos-99-00 | MOSSA | 89 | 1 | collective | I | C | IND | 20–40 | MA |
| 73 | Fm-Mos-99-00 | MOSSA | 89 | 2 | collective | I | C | M(?) | 20+ | GA |
| 74 | Fm-Mos-99-00 | MOSSA | 89 | 3 | collective | I | C | M(?) | 40+ | OA |
| 75 | Fm-Mos-99-00 | MOSSA | 89 | 4 | collective | I | C | IND | 5–10 | OC |
| 76 | Fm-Mos-99-01 | MOSSA | 91 | 1 | double | I | C | M | 20–40 | MA |
| 77 | Fm-Mos-99-02 | MOSSA | 91 | 2 | double | I | C | M | 40+ | OA |
| 78 | Fm-Mos-99-00 | MOSSA | 95 | 1 | single | C | B | M | 20–40 | MA |
| 79 | Fm-Mis-1959 (Alidori) | MISERICORDIA | 3 | Busta  1 and 2 | single | C | A | F(?) | 20+ | GA |
| 80 | Fm-Mis-1956 (?) | MISERICORDIA | 3 | 1 | double | C | B | F | 40+ | OA |
| 81 | Fm-Mis-1956 (?) | MISERICORDIA | 3 | 2 | double | C | B | M | 20–40 | MA |
| 82 | Fm-Mis-1956 (Brusadin) | MISERICORDIA | 3 | 1 | double | I | B | F(?) | 20–40 | MA |
| 83 | Fm-Mis-1956 (Brusadin) | MISERICORDIA | 3 | 2 | double | I | B | M(?) | 40+ | OA |
| 84 | Fm-Mis-1956 (Bonfigli) | MISERICORDIA | 4 | 1 | single | C | IND | M(?) | 20+ | GA |
| 85 | Fm-Mis-1956 (Brusadin) | MISERICORDIA | 4 | 1 | single | C | IND | IND | 1–5 | YC |
| 86 | Fm-Mis-1956 (Bonfigli) | MISERICORDIA | 6 | 1 | single | C | IND | IND | 1–5 | YC |
| 87 | Fm-Mis-1959 (Alidori) | MISERICORDIA | 6 | 1 | double | C | IND | IND | 1–5 | YC |
| 88 | Fm-Mis-1959 (Alidori) | MISERICORDIA | 6 | 2 | double | C | IND | IND | 1–5 | YC |
| 89 | Fm-Mis-1956 (Bonfigli) | MISERICORDIA | 7 | 1 | single | C | IND | M | 20+ | GA |
| 90 | Fm-Mis-1956 (Brusadin) | MISERICORDIA | 7 | 1 | single | I | B | M | 20–40 | MA |
| 91 | Fm-Mis-1956 (Bonfigli) | MISERICORDIA | 9 | 1 | single | C | IND | IND | 20+ | GA |
| 92 | Fm-Mis-1959 (Alidori) | MISERICORDIA | 9 | 1 | single | C | IND | F | 20+ | GA |
| 93 | Fm-Mis-1956 (Bonfigli) | MISERICORDIA | 11 | 1 | single | C | B | F | 40+ | OA |
| 94 | Fm-Mis-1959 (Alidori) | MISERICORDIA | 13 | 1 | single | C | A | IND | 20+ | GA |
| 95 | Fm-Mis-1956 (Bonfigli) | MISERICORDIA | 16 | 1 | single | I | C | IND | 20+ | GA |
| 96 | Fm-Mis-1959 (Alidori) | MISERICORDIA | 17A | 1 | single | C | A | F(?) | 20+ | GA |
| 97 | Fm-Mis-? | MISERICORDIA | 18 | 1 | single | C | B | M(?) | 20+ | GA |
| 98 | Fm-Mis-1956 (Bonfigli) | MISERICORDIA | 20 | 1 | single | C | B | M | 20+ | GA |
| 99 | Fm-Mis-1956 (Bonfigli) | MISERICORDIA | 22 | 1 | single | C | B | F(?) | 20–40 | MA |
| 100 | Fm-Mis-1959 (Alidori) | MISERICORDIA | 22 | 1 | single | C | B | M | 20+ | GA |
| 101 | Fm-Mis-? | MISERICORDIA | 23 | 1 | single | C | IND | M | 20+ | GA |
| 102 | Fm-Mis-1956 (Bonfigli) | MISERICORDIA | 27 | 1 | single | C | A | IND | 20+ | GA |
| 103 | Fm-Mis-1959 (Alidori) | MISERICORDIA | 29 | 1 | single | C | IND | F | 20–40 | MA |
| 104 | Fm-Mis-1956 (Bonfigli) | MISERICORDIA | 32 | 1 | single | C | A | M | 20+ | GA |
| 105 | Fm-Mis-1956 (Bonfigli) | MISERICORDIA | 34 | 1 | single | C | A | F | 20–40 | MA |
| 106 | Fm-Mis-1956 (Bonfigli) | MISERICORDIA | 36 | 1 | single | C | A | M(?) | 40+ | OA |
| 107 | Fm-Mis-1956 (Bonfigli?) | MISERICORDIA | 37 | 1 | single | C | IND | IND | 20+ | GA |
| 108 | Fm-Mis-1956 (Bonfigli) | MISERICORDIA | 38 | 1 | single | C | A(?) | M(?) | 20+ | GA |
| 109 | Fm-Mis-1956 (Bonfigli) | MISERICORDIA | 40 | 1 | single | C | IND | M | 20–40 | MA |
| 110 | Fm-Mis-1956 (Bonfigli) | MISERICORDIA | 43 | 1 | single | C | B | F(?) | 40+ | OA |
| 111 | Fm-Mis-1956 (Bonfigli) | MISERICORDIA | 44 | 1 | single | C | B | F | 20–40 | MA |
| 112 | Fm-Mis-1956 (Bonfigli) | MISERICORDIA | 45 | 1 | single | C | A | F | 20–40 | MA |
| 113 | Fm-Mis-1956 (Bonfigli) | MISERICORDIA | 46 | 1 | single | C | A | F | 15–20 | YA |
| 114 | Fm-Mis-1956 (Bonfigli) | MISERICORDIA | 47 | 1 | single | C | A | F | 20–40 | MA |
| 115 | Fm-Mis-1956 (Bonfigli) | MISERICORDIA | 48 | 1 | single | C | A | F | 20+ | GA |
| 116 | Fm-Mis-1956 (Bonfigli) | MISERICORDIA | 49 | 1 | single | C | A(?) | F | 40+ | OA |
| 117 | Fm-Mis-1956 (Bonfigli) | MISERICORDIA | 52 | 1 | single | C | A | IND | 5–10 | OC |
| 118 | Fm-Mis-1956 (Brusadin) | MISERICORDIA | 95 | 1 | single | I | A | IND | 1–5 | YC |
| 119 | Fm-Mis-1956 (Brusadin) | MISERICORDIA | 116_1 | 1 | double | I | B | M | 40+ | OA |
| 120 | Fm-Mis-1956 (Brusadin) | MISERICORDIA | 116_2 | 2 | double | I | B | M | 15–20 | YA |

Table S3. Summary of the analysed tombs divided by chronology and ritual. Chron: A = end of the 9^th^ century to the beginning of the 8^th^ century BCE; A(?) = probable end of the 9^th^ century to the beginning of the 8^th^ century BCE; B = second half of the 8^th^ century BCE; C = 7^th^ and 6^th^ century BCE; IND = indeterminate.

| **Chron** | **Cremations** | **Inhumations** | **Grand Total** |
| --- | --- | --- | --- |
| A and A(?) | 14 | 1 | 15 |
| B | 21 | 22 | 43 |
| C | - | 45 | 45 |
| IND | 12 | 5 | 17 |
| **Grand Total** | **47** | **73** | **120** |

Table S4. δ^13^C, δ^15^N and C:N atomic values (C:N_a_). Sex: F = female; F(?) = probable female; M = male; M(?) = probable male; IND = indeterminate. Age: YC (young child) = 1–5 years of age; OC (older child) = 5–10 years of age; J (juvenile) = 10–15 years of age; A (adult) = 16+ years of age.

| **n** | **excavation** | **tomb**  **id** | **ind**  **id** | **sex** | **age**  **group** | **lab**  **id** | **sample** | **δ^13^C** **VPDB (**‰) | **δ^15^N**  **AIR**  **(**‰) | **C:N_a_** | | **collagen yield%** | |
| --- | --- | --- | --- | --- | --- | --- | --- | --- | --- | --- | --- | --- | --- |
| 1 | Fm-Mos-99/00 | 5 | 1 | M | A | FM001 | rib | -20.01 | 7.93 | 3.16 | 6.2 | |  |
| 2 | Fm-Mos-99/00 | 7 | 1 | M | A | FM002 | rib | FAIL | FAIL | FAIL | FAIL | |  |
| 3 | Fm-Mos-99/00 | 10 | 1 | F(?) | A | FM003 | rib | -19.45 | 8.25 | 3.17 | 3.6 | |  |
| 4 | Fm-Mos-99/00 | 17 | 1 | M | A | FM004 | rib | -19.94 | 8.19 | 3.16 | 5.3 | |  |
| 5 | Fm-Mos-99/00 | 29 | 1 | IND | OC | FM005 | rib | -20.16 | 7.19 | 3.15 | 6.1 | |  |
| 6 | Fm-Mos-99/00 | 51 | 1 | M | A | FM006 | rib | -20.12 | 8.1 | 3.17 | 2.6 | |  |
| 7 | Fm-Mos-99/00 | 58 | 1 | IND | A | FM007 | rib | -19.84 | 8.94 | 3.15 | 0.8 | |  |
| 8 | Fm-Mos-99/00 | 58 | 2 | IND | OC | FM008 | rib | FAIL | FAIL | FAIL | FAIL | |  |
| 9 | Fm-Mos-99/00 | 62 | 1 | IND | OC | FM009 | rib | FAIL | FAIL | FAIL | FAIL | |  |
| 10 | Fm-Mos-99/00 | 64 | 1 | M | A | FM010 | rib | FAIL | FAIL | FAIL | FAIL | |  |
| 11 | Fm-Mos-99/00 | 65 | 1 | IND | J | FM011 | rib | FAIL | FAIL | FAIL | FAIL | |  |
| 12 | Fm-Mos-99/00 | 65 | 2 | IND | OC | FM012 | rib | FAIL | FAIL | FAIL | FAIL | |  |
| 13 | Fm-Mos-99/00 | 67 | 1 | M | A | FM013 | rib | FAIL | FAIL | FAIL | FAIL | |  |
| 14 | Fm-Mos-99/00 | 70 | 1 | IND | OC | FM014 | rib | -20.01 | 7.85 | 3.14 | 3.9 | |  |
| 15 | Fm-Mos-99/00 | 81 | 5 | IND | OC | FM015 | rib | -20.09 | 8.05 | 3.12 | 3.7 | |  |
| 16 | Fm-Mos-99/00 | 81 | 6 | M | A | FM016 | rib | -20.03 | 7.97 | 3.12 | 3.6 | |  |
| 17 | Fm-Mos-99/00 | 81 | 7 | M | A | FM017 | rib | -20.09 | 7.92 | 3.12 | 7.3 | |  |
| 18 | Fm-Mos-99/00 | 81 | 8 | F | A | FM018 | rib | -19.72 | 8.03 | 3.11 | 4.1 | |  |
| 19 | Fm-Mos-99/00 | 81 | 11 | M | A | FM019 | rib | -19.84 | 8.62 | 3.14 | 2.3 | |  |
| 20 | Fm-Mos-99/00 | 81 | 12 | F | A | FM020 | rib | -19.67 | 8.23 | 3.11 | 1.5 | |  |
| 21 | Fm-Mos-99/00 | 81 | 13 | F | A | FM021 | rib | -20.03 | 7.81 | 3.15 | 4.6 | |  |
| 22 | Fm-Mos-99/00 | 81 | 14 | M | A | FM022 | rib | -19.68 | 8.32 | 3.14 | 4.4 | |  |
| 23 | Fm-Mos-99/00 | 88 | 1 | M | A | FM023 | rib | FAIL | FAIL | FAIL | FAIL | |  |
| 24 | Fm-Mos-99/00 | 88 | 2 | IND | A | FM024 | rib | FAIL | FAIL | FAIL | FAIL | |  |
| 25 | Fm-Mis-1956(Bonfigli) | 95 | 1 | IND | YC | FM025 | rib | -19.47 | 7.46 | 3.13 | 1.8 | |  |

Table S5. δ^13^C, δ^15^N and C:N atomic values (C:N_a_) values of fauna remains – cattle, deer, pig, sheep/goat – from Coppa Nevigata. Fermo δ^13^C and δ^15^N baselines derive from paleo(environmental) studies conducted on the closest available site to Fermo known in the scholarly literature, namely Coppa Nevigata ^68^.

| **sample id** | **specimen** | **chron** | **site** | **δ^13^C** **VPDB (**‰) | **δ^15^N**  **AIR**  **(**‰) | **C:N_a_** | **%C** | **%N** | **collagen yield%** | **reference** |
| --- | --- | --- | --- | --- | --- | --- | --- | --- | --- | --- |
| CNF1 | cattle | Bronze age | Coppa Nevigata | -21.21 | 5.43 | 3.14 | 41.40 | 15.40 | 12.61 | Miller 2018 |
| CNF2 | cattle | Bronze age | Coppa Nevigata | -20.14 | 7.72 | 3.3 | 10.88 | 3.85 | 5.33 | Miller 2018 |
| CNF3 | cattle | Bronze age | Coppa Nevigata | -20.51 | 6.37 | 3.21 | 20.58 | 7.47 | 6.45 | Miller 2018 |
| CNF4 | cattle | Bronze age | Coppa Nevigata | -20.62 | 6.58 | 3.23 | 31.22 | 11.29 | 7.49 | Miller 2018 |
| CNF5 | cattle | Not specified | Coppa Nevigata | -20.53 | 5.63 | 3.24 | 19.03 | 6.86 | 4.58 | Miller 2018 |
| CNF6 | cattle | Bronze age | Coppa Nevigata | -20.63 | 6.52 | 3.2 | 24.14 | 8.79 | 3.11 | Miller 2018 |
| CNF7 | cattle | Not specified | Coppa Nevigata | -21.18 | 3.38 | 3.14 | 47.97 | 17.81 | 16.28 | Miller 2018 |
| CNF8 | cattle | Not specified | Coppa Nevigata | -20.85 | 5.02 | 3.14 | 25.83 | 9.61 | 10.68 | Miller 2018 |
| CNF17 | deer | Bronze age | Coppa Nevigata | -20.27 | 5.18 | 3.15 | 24.61 | 9.11 | 10.76 | Miller 2018 |
| CNF18 | deer | Bronze age | Coppa Nevigata | -21.23 | 6.86 | 3.27 | 2.13 | 0.76 | 8.97 | Miller 2018 |
| CNF19 | deer | Bronze age | Coppa Nevigata | -19.66 | 7.09 | 3.19 | 38.47 | 14.09 | 9.81 | Miller 2018 |
| CNF20 | deer | Not specified | Coppa Nevigata | -21.23 | 5.57 | 3.22 | 31.81 | 11.52 | 10.21 | Miller 2018 |
| CNF22 | deer | Not specified | Coppa Nevigata | -20.47 | 6.97 | 3.16 | 34.87 | 12.86 | 13.90 | Miller 2018 |
| CNF29 | deer | Not specified | Coppa Nevigata | -18.87 | 8.5 | 3.15 | 41.45 | 15.34 | 12.23 | Miller 2018 |
| CNF9 | pig | Bronze age | Coppa Nevigata | -19.85 | 6.03 | 3.12 | 35.75 | 13.37 | 9.03 | Miller 2018 |
| CNF10 | pig | Bronze age | Coppa Nevigata | -20.63 | 4.97 | 3.19 | 20.94 | 7.67 | 9.99 | Miller 2018 |
| CNF11 | pig | Bronze age | Coppa Nevigata | -20.37 | 4.25 | 3.23 | 24.47 | 8.85 | 9.10 | Miller 2018 |
| CNF12 | pig | Not specified | Coppa Nevigata | -20.27 | 6.29 | 3.23 | 30.32 | 10.94 | 4.94 | Miller 2018 |
| CNF13 | pig | Not specified | Coppa Nevigata | -20.71 | 4.1 | 3.18 | 18.05 | 6.62 | 11.53 | Miller 2018 |
| CNF14 | pig | Bronze age | Coppa Nevigata | -20.6 | 7.15 | 3.20 | 27.22 | 9.91 | 12.44 | Miller 2018 |
| CNF15 | pig | Not specified | Coppa Nevigata | -20.31 | 8.69 | 3.14 | 26.78 | 9.95 | 11.82 | Miller 2018 |
| CNF16 | pig | Bronze age | Coppa Nevigata | -20.44 | 6.68 | 3.30 | 37.95 | 13.95 | 12.05 | Miller 2018 |
| CNF23 | sheep/goat | Bronze age | Coppa Nevigata | -19.39 | 7.23 | 3.16 | 38.34 | 14.16 | 8.82 | Miller 2018 |
| CNF24 | sheep/goat | Bronze age | Coppa Nevigata | -19.94 | 5.87 | 3.0 | 25.76 | 9.26 | 7.44 | Miller 2018 |
| CNF26 | sheep/goat | Bronze age | Coppa Nevigata | -20.69 | 7.64 | 3.15 | 41.06 | 15.19 | 12.12 | Miller 2018 |
| CNF27 | sheep/goat | Not specified | Coppa Nevigata | -20.44 | 6.69 | 3.16 | 42.42 | 15.64 | 8.03 | Miller 2018 |
| CNF28 | sheep/goat | Not specified | Coppa Nevigata | -19.94 | 7.29 | 3.17 | 33.38 | 12.30 | 17.65 | Miller 2018 |

Table S6. Baseline samples (n = 11) and results for Fermo. The “same” column indicates samples collected in the same area. Errors (±95% c.l.) of ^87^Sr/^86^Sr analyses represent the reproducibility of the SRM987 standard at the time of analysis.

| **n** | **lab id** | **period** | **sample** | **87Sr/86Sr** | **±95% c.l.** | **km** | **same** | **North_coordinate** | **East_coordinate** |
| --- | --- | --- | --- | --- | --- | --- | --- | --- | --- |
| 1 | FM-SR-WATER2 | modern | water | 0.709075 | 0.000022 | 0–7 | 1 | 43°12'3.15719'' | 13°46'41.64466'' |
| 2 | FM-SR-WATER1 | modern | water | 0.709108 | 0.000022 | 0–7 | 2 | 43°5'57'' | 13°46'11'' |
| 3 | FM-SR-SOIL4 | modern | soil | 0.708682 | 0.000017 | 0–7 | 2 | 43°5'57'' | 13°46'11'' |
| 4 | FM-SR-SOIL5 | modern | soil | 0.708797 | 0.000017 | 0–7 | 1 | 43°11'29'' | 13°46'4'' |
| 5 | FM-SR-SOIL3 | archaeological | soil | 0.708747 | 0.000017 | Fermo | 3 | 43°10'20.865'' | 13°44'30.522'' |
| 6 | FM-SR-SNAIL2 | archaeological | snail | 0.708530 | 0.000017 | Fermo | 3 | 43°10'20.865'' | 13°44'30.522'' |
| 7 | FM-SR-SNAIL3 | archaeological | snail | 0.708561 | 0.000017 | Fermo | 3 | 43°10'20.865'' | 13°44'30.522'' |
| 8 | FM-SR-FAUNA3 | archaeological | enamel  (*sus*) | 0.708706 | 0.000017 | Fermo | 3 | 43°09'49.01'' | 13°43'28.80'' |
| 9 | FM-SR-FAUNA2 | archaeological | enamel  (*ovis*) | 0.708744 | 0.000017 | Fermo | 3 | 43°09'49.01'' | 13°43'28.80'' |
| 10 | FM-SR-GRASS3 | modern | grass | 0.709177 | 0.000022 | 0–7 | 1 | 43°11'29.02657" | 13°46'8.84942'' |
| 11 | FM-SR-GRASS1 | modern | grass | 0.709144 | 0.000022 | 0–7 | 4 | 43°8'24.500411" | 13°48'44.53204" |

Table S7. List of ^87^Sr/^86^Sr results for human individuals with archaeological and osteological information. Ritual: C = cremation; I = inhumation. Sample type: PB = petrous bone; E = enamel. Chron: A = end of the 9^th^ century to the beginning of the 8^th^ century BCE; A(?) = probable end of the 9^th^ century to the beginning of the 8^th^ century BCE; B = second half of the 8^th^ century BCE; C = 7^th^ and 6^th^ century BCE; IND = indeterminate. Sex: F = female; F(?) = probable female; M = male; M(?) = probable male; SA = subadult; IND = indeterminate. Age: YC (young child) = 1–5 years of age; OC (older child) = 5–10 years of age; J (juvenile) = 10–15 years of age; A (adult) = 16+ years of age. Errors (±95% c.l.) of ^87^Sr/^86^Sr analyses represent the reproducibility of the SRM987 standard at the time of analy­sis.

| **n** | **excavation** | **tomb**  **id** | **ind**  **id** | **ritual** | **chron** | **sex** | **age** | **lab**  **id** | **sample** | **^87^Sr/^86^Sr** | **±95% c.l.** | **FTIR** |
| --- | --- | --- | --- | --- | --- | --- | --- | --- | --- | --- | --- | --- |
| 1 | Fm-Mis-1956  (Bonfigli) | 34 | 1 | C | A | F | A | FM-SR-01 | RPB | 0.709177 | 0.000017 | femur |
| 2 | Fm-Mis-1956  (Bonfigli) | 36 | 1 | C | A | M(?) | A | FM-SR-02 | RPB | 0.709168 | 0.000023 |  |
| 3 | Fm-Mis-1956  (Bonfigli) | 43 | 1 | C | B | F(?) | A | FM-SR-03 | LPB | 0.708986 | 0.000017 |  |
| 4 | Fm-Mis-1956  (Bonfigli) | 44 | 1 | C | B | F | A | FM-SR-04 | LPB | 0.708932 | 0.000017 |  |
| 5 | Fm-Mis-1956  (Bonfigli) | 45 | 1 | C | A | F | A | FM-SR-05 | LPB | 0.708954 | 0.000017 |  |
| 6 | Fm-Mis-1956  (Bonfigli) | 40 | 1 | C | IND | M | A | FM-SR-06 | LPB | 0.709020 | 0.000017 |  |
| 7 | Fm-Mos-99/00 | 56 | 1 | C | B | IND | J | FM-SR-07 | LPB | 0.709091 | 0.000017 |  |
| 8 | Fm-Mos-99/00 | 63 | 1 | C | B | M | A | FM-SR-09 | LPB | 0.708945 | 0.000017 |  |
| 9 | Fm-Mis-1959  (Alidori) | 3 | Busta  1 and 2 | C | A | F(?) | A | FM-SR-11 | RPB | 0.709083 | 0.000024 | petrous |
| 10 | Fm-Mis-1956  (Bonfigli) | 20 | 1 | C | B | M | A | FM-SR-12 | RPB | 0.709154 | 0.000017 |  |
| 11 | Fm-Mos-99/00 | 59D | 1 | C | B | F | A | FM-SR-13 | RPB | 0.709678 | 0.000017 | femur |
| 12 | Fm-Mos-99/00 | 59A | 1 | C | B | M | A | FM-SR-14 | LPB | 0.709528 | 0.000017 | petrous |
| 13 | Fm-Mos-99/00 | 58 | 3 | C | B | M | A | FM-SR-15 | LPB | 0.709081 | 0.000017 |  |
| 14 | Fm-Mos-99/00 | 58 | 2 | I | B | IND | OC | FM-SR-16 | LRM1 | 0.708880 | 0.000022 |  |
| 15 | Fm-Mos-99/00 | 58 | 1 | I | B | IND | A | FM-SR-17 | URM1 | 0.708932 | 0.000022 |  |
| 16 | Fm-Mos-99/00 | 21 | 1 | C | B | F | A | FM-SR-18 | RPB | 0.708945 | 0.000017 |  |
| 17 | Fm-Mos-99/00 | 31 | 1 | C | B | F | A | FM-SR-19 | LPB | 0.708955 | 0.000017 |  |
| 18 | Fm-Mos-99/00 | 95 | 1 | C | B | M | A | FM-SR-20 | RPB | 0.709192 | 0.000017 | petrous |
| 19 | Fm-Mis-1959  (Alidori) | 22 | 1 | C | B | M | A | FM-SR-21 | LPB | 0.708943 | 0.000017 |  |
| 20 | Fm-Mis1956  (Brusadin) | 4 | 1 | C | IND | IND | YC | FM-SR-23 | LPB | 0.708934 | 0.000017 |  |
| 21 | Fm-Mis-1959  (Alidori) | 29 | 1 | C | IND | F | A | FM-SR-24 | LPB | 0.708982 | 0.000017 |  |
| 22 | Fm-Mos-1968 | 11 | 2 | I | B | F | A | FM-SR-25 | ULM2(?) | 0.708879 | 0.000022 |  |
| 23 | Fm-Mos-1968 | 11 | 3 | I | B | IND | J | FM-SR-26 | ULM1 | 0.708906 | 0.000022 |  |
| 24 | Fm-Mos-1968 | 11 | 4 | I | B | IND | YC | FM-SR-27 | LLM1 | 0.708916 | 0.000022 |  |
| 25 | Fm-Mos-1968 | 11 | 5 | C | B | F(?) | A | FM-SR-28 | RPB | 0.709202 | 0.000022 |  |
| 26 | Fm-Mos-99/00 | 29 | 1 | I | C | IND | OC | FM-SR-29 | ULM1 | 0.708890 | 0.000022 |  |
| 27 | Fm-Mis-1956  (Brusadin) | 7 | 1 | I | B | M | A | FM-SR-30 | LLM1 | 0.708900 | 0.000022 |  |
| 28 | Fm-Mos-99/00 | 64 | 1 | I | B | M | A | FM-SR-31 | LLM2 | 0.708842 | 0.000022 |  |
| 29 | Fm-Mos-99/00 | 78 | 1 | C | B | F(?) | A | FM-SR-32 | RPB | 0.709517 | 0.000022 |  |
| 30 | Fm-Mos-99/00 | 67 | 1 | I | C | M | A | FM-SR-33 | ULM1 | 0.708831 | 0.000022 |  |
| 31 | Fm-Mos-99/00 | 67 | 3 | I | B | M | A | FM-SR-34 | LRM1 | 0.708858 | 0.000022 |  |
| 32 | Fm-Mos-99/00 | 10 | 1 | I | IND | F(?) | A | FM-SR-35 | LRM1 | 0.708970 | 0.000022 |  |
| 33 | Fm-Mos-99/00 | 62 | 1 | I | B | IND | OC | FM-SR-36 | URM1 | 0.708896 | 0.000022 |  |
| 34 | Fm-Mos-99/00 | 62 | 2 | I | B | IND | A | FM-SR-37 | URM1 | 0.708949 | 0.000022 |  |
| 35 | Fm-Mos-99/00 | 65 | 1 | I | B | IND | J | FM-SR-38 | ULM1 | 0.708819 | 0.000022 |  |
| 36 | Fm-Mos-99/00 | 65 | 2 | I | B | IND | OC | FM-SR-39 | LLM1 | 0.708825 | 0.000022 |  |
| 37 | Fm-Mis-1956  (Bonfigli) | 22 | 1 | C | B | F(?) | A | FM-SR-40 | M3 | 0.708960 | 0.000022 |  |
| 38 | Fm-Mis-1956  (Bonfigli) | 49 | 1 | C | A(?) | F | A | FM-SR-42 | LPB | 0.709031 | 0.000022 |  |
| 39 | Fm-Mis-1956  (Bonfigli) | 52 | 1 | C | A | IND | OC | FM-SR-44 | RPB | 0.709079 | 0.000022 |  |
| 40 | Fm-Mos-99/00 | 89 | 1 | I | C | IND | A | FM-SR-45 | ULM1 | 0.708874 | 0.000022 |  |
| 41 | Fm-Mos-99/00 | 89 | 3 | I | C | M(?) | A | FM-SR-46 | URM1 | 0.708932 | 0.000022 |  |
| 42 | Fm-Mos-99/00 | 89 | 4 | I | C | IND | OC | FM-SR-47 | ULM1 | 0.708951 | 0.000022 |  |
| 43 | Fm-Mos-99/00 | 17 | 1 | I | B | M | A | FM-SR-48 | ULM1 | 0.709581 | 0.000022 |  |
| 44 | Fm-Mos-99/00 | 51 | 1 | I | B | M | A | FM-SR-49 | ULM1 | 0.708933 | 0.000022 |  |
| 45 | Fm-Mis-1956  (Brusadin) | 95 | 1 | I | A | IND | YC | FM-SR-50 | URdm2 | 0.708880 | 0.000022 |  |
| 46 | Fm-Mos-1968 | 2 | 4 | I | C | IND | OC | FM-SR-52 | LLM1 | 0.708984 | 0.000020 |  |
| 47 | Fm-Mos-99-00 | 81 | 13 | I | C | F | A | FM-SR-53 | ULM1 | 0.708884 | 0.000020 |  |
| 48 | Fm-Mos-99-00 | 81 | 5 | I | C | IND | OC | FM-SR-54 | LRm1 | 0.708960 | 0.000020 |  |
| 49 | Fm-Mos-99-00 | 81 | 11 | I | C | M | A | FM-SR-55 | LRM1 | 0.708901 | 0.000020 |  |
| 50 | Fm-Mos-99-00 | 81 | 12 | I | C | F | A | FM-SR-56 | ULM1 | 0.708880 | 0.000020 |  |
| 51 | Fm-Mos-99-00 | 81 | 14 | I | C | M | A | FM-SR-57 | ULM1 | 0.708889 | 0.000020 |  |
| 52 | Fm-Mos-99-00 | 88 | 1 | I | C | M | A | FM-SR-58 | URM1 | 0.708911 | 0.000020 |  |
| 53 | Fm-Mos-99-00 | 88 | 2 | I | C | IND | A | FM-SR-59 | LLM1 | 0.708878 | 0.000020 |  |
| 54 | Fm-Mos-99-00 | 88 | 3 | I | C | M(?) | A | FM-SR-60 | URM1 | 0.708922 | 0.000020 |  |

Table S8. Basic statistical consideration on ^87^Sr/^86^Sr values for human samples from Fermo.

| Descriptive statistics for ^87^Sr/^86^Sr outcomes at Fermo | |
| --- | --- |
| mean | 0.70900 |
| median | 0.70894 |
| min | 0.70882 |
| max | 0.70968 |
| sd | 0.00019 |
| tot | 54 |

# **References**

1. Bartoloni, G. *La cultura villanoviana all’inizio della storia Etrusca* (Carocci, 2002).

2. Iaia, C. *Simbolismo funerario e ideologia alle origini di una civiltà urbana: forme rituali nelle sepolture “villanoviane” a Tarquinia e Vulci, e nel loro entroterra* (All’Insegna del Giglio, 1999).

3. Pacciarelli, M. *Dal villaggio alla città: la svolta protourbana del 1000 a.C. nell’Italia tirrenica* (All’Insegna del Giglio, 2000).

4. Peroni, R. Formazione e sviluppo dei centri protourbani medio tirrenici in *L’Italia alle soglie della storia* (ed. Peroni, R.) 26–31 (Laterza, 2000).

5. Cerchiai, L. L’identità etnica come processo di relazione: alcune riflessioni a proposito del mondo italico in *Le origini degli Etruschi: Storia, archeologia, antropologia* (ed. Bellelli, V.) 345–357 (L’Erma di Bretschneider, 2012).

6. Stoddart, S. Power and place in Etruria in *Eurasia at the dawn of history* (eds. Fernandez-Gotz, M. & Krausse, D.) 304–318 (Cambridge University Press, 2017). https://doi.org/10.1017/9781316550328.021.

7. Cinquantaquattro, T. & Pellegrino, C. Southern Campania in *Etruscology* (ed. Naso, A.) 1359–1394 (De Gruyter, 2017).

8. Malnati, L. Emilia in *Etruscology* (ed. Naso, A.) 1437–1452 (De Gruyter, 2017).

9. von Eles, P. & Baldelli, G. Romagna and the Marches in *Etruscology* (ed. Naso, A.) 1453–1500 (De Gruyter, 2017).

10. Pohl, I. *The Iron Age necropolis of Sorbo at Cerveteri* (Astrom, 1972).

11. Iaia, C. & Pacciarelli, M. La cremazione in area mediotirrenica tra Bronzo Finale e Primo Ferro in *Les necròpolis d’incineració entre l’Ebre i el Tíber (segles IX–VI aC): metodologia, pràctiques funeràries i societat* (eds. Rovira Hortalà, M. C., López Cachero, F. J. & Mazière, F.) 341–355 (Mac, 2012).

12. Peroni, R. Villanoviano a Fermo? in *La civiltà picena nelle Marche: studi in onore di Giovanni Annibaldi. Ancona, 10–13 luglio 1988* (ed. Dardari, M.) 13–38 (Maroni, 1992).

13. Peroni, R. Variazioni sul tema del concetto di “Villanoviano” applicato alla Campania in *La presenza etrusca nella Campania meridionale: atti delle giornate di studio. Salerno–Pontecagnano, 16–18 novembre 1990* (eds. Gastaldi, P. & Maetzke, G.) 37–48 (Olschki, 1994).

14. d’Agostino, B. Gli Etruschi e gli altri nella Campania settentrionale in *Atti del XXVI convegno di studi Etruschi ed Italici. Caserta–Santa Maria Capua Vetere–Capua- Teano, 11–15 novembre 2007* (eds. Paoletti, O. & Bettini, M. C.) 69–91 (Fabrizio Serra Editore, 2011).

15. van der Merwe, N. J. & Vogel, J. C. 13C content of human collagen as a measure of prehistoric diet in woodland North America. *Nature* **276**, 815–816 (1978).

16. Ambrose, S. H. Isotopic Analysis of paleodiets: methodological and interpretive considerations in *Investigations of ancient human tissue: chemical analyses in anthropology* (ed. Sandford, M. K.) 59–130 (1993).

17. Jim, S., Ambrose, S. H. & Evershed, R. P. Stable carbon isotopic evidence for differences in the dietary origin of bone cholesterol, collagen and apatite: implications for their use in palaeodietary reconstruction. *Geochim. Cosmochim. Acta* **68**, 61–72 (2004).

18. Lai, L. *et al.* Interpreting stable isotopic analyses: case studies on Sardinian prehistory in *Archaeological chemistry: analytical techniques and archaeological interpretation* (eds. Glascock, M. D., Speakman, R. J. & Popelka-Filcoff, R. S.) 114–136 (American Chemical Society, 2007). https://doi.org/10.1021/bk-2007-0968.ch006.

19. Lai, L. *et al.* Diet in the Sardinian Bronze Age: models, collagen isotopic data, issues and perspectives. *Préhistoires Méditerranéennes* [Online 4], (2013).

20. Snoeck, C., Schulting, R. J., Lee-Thorp, J. A., Lebon, M. & Zazzo, A. Impact of heating conditions on the carbon and oxygen isotope composition of calcined bone. *J. Archaeol. Sci.* **65**, 32–43 (2016).

21. Brown, T. & Brown, K. *Biomolecular archaeology: an introduction*. (John Wiley & Sons, 2011).

22. Malainey, M. E. *A consumer’s guide to archaeological science* (Springer, 2011).

23. Lee-Thorp, J. A. On isotopes and old bones. *Archaeometry* **50**, 925–950 (2008).

24. DeNiro, M. J. & Epstein, S. Influence of diet on the distribution of nitrogen isotopes in animals. *Geochim. Cosmochim. Acta* **45**, 341–351 (1981).

25. Schoeninger, M. J. & DeNiro, M. J. Nitrogen and carbon isotopic composition of bone collagen from marine and terrestrial animals. *Geochim. Cosmochim. Acta* **48**, 625–639 (1984).

26. Sealy, J. C., van der Merwe, N. J., Thorp, J. A. L. & Lanham, J. L. Nitrogen isotopic ecology in southern Africa: implications for environmental and dietary tracing. *Geochim. Cosmochim. Acta* **51**, 2707–2717 (1987).

27. Richards, M. A brief review of the archaeological evidence for Palaeolithic and Neolithic subsistence. *Eur. J. Clin. Nutr.* **56**, 1270–1278 (2002).

28. Hedges, R. E. M., Clement, J. G., Thomas, C. D. & O’Connell, T. Collagen turnover in the adult femoral mid-shaft: modelled from anthropogenic radiocarbon tracer measurements. *Am. J. Phys. Anthropol.* **133**, 808–816 (2007).

29. Tafuri, M. A., Craig, O. E. & Canci, A. Stable isotope evidence for the consumption of millet and other plants in Bronze Age Italy. *Am. J. Phys. Anthropol.* **139**, 146–153 (2009).

30. Katzenberg, M. A. & Harrison, R. G. What’s in a bone? Recent advances in archaeological bone chemistry. *Journal of Archaeological Research* **5**, 265–293 (1997).

31. Craig, O. E. *et al.* Stable isotopic evidence for diet at the Imperial Roman coastal site of Velia (1st and 2nd Centuries AD) in Southern Italy. *Am. J. Phys. Anthropol.* **139**, 572–583 (2009).

32. Hobson, K. A. & Clark, R. G. Assessing avian diets using stable isotopes II: factors influencing diet-tissue fractionation. *Condor* **94,** 189–197 (1992).

33. Fuller, B. T. *et al.* Nitrogen balance and δ15N: why you’re not what you eat during nutritional stress. *Rapid Communications in Mass Spectrometry* **19**, 2497–2506 (2005).

34. Schoeninger, M., DeNiro, M. & Tauber, H. Stable nitrogen isotope ratios of bone collagen reflect marine and terrestrial components of prehistoric human diet. *Science (1979)* **220**, 1381–1383 (1983).

35. Cox, G. & Sealy, J. C. Investigating identity and life histories: isotopic analysis and historical documentation of slave skeletons found on the Cape Town Foreshore, South Africa. *Int. J. Hist. Archaeol.* **1**, 207-224. (1997).

36. Bentley, A. Strontium isotopes from the earth to the archaeological skeleton: a review. *J. Archaeol. Method. Theory* **13**, 135–187 (2006).

37. Sengeløv, A. *et al.* Understanding the post-Archaic population of Satricum, Italy: a bioarchaeological approach. *J. Archaeol. Sci. Rep.* **31**, 102285 (2020).

38. Kutschera, W. & Müller, W. “Isotope language” of the Alpine Iceman investigated with AMS and MS. *Nucl. Instrum. Methods Phys. Res. B* **204**, 705–719 (2003).

39. Price, T. D., Burton, J. H. & Bentley, R. A. The characterization of biologically available strontium isotope ratios for the study of prehistoric migration. *Archaeometry* **44**, 117–135 (2002).

40. Grimstead, D. N., Nugent, S. & Whipple, J. Why a standardization of strontium isotope baseline environmental data is needed and recommendations for methodology. *Advances in Archaeological Practice* **5**, 1–12 (2017).

41. Veselka, B. *et al.* Strontium isotope ratios related to childhood mobility: revisiting sampling strategies of the calcined human pars petrosa ossis temporalis. *Rapid Communications in Mass Spectrometry* (2020), <https://doi.org/10.1002/rcm.9038>.

42. Sabel, N. *et al.* Neonatal lines in the enamel of primary teeth: a morphological and scanning electron microscopic investigation. *Arch. Oral Biol.* **53**, 954–963 (2008).

43. Liversidge, H. M. The dentition in *Developmental juvenile osteology* (eds. Scheuer, C., Black, S. & Cunningham, L.) 149-176 (Academic Press, 2016).

44. AlQahtani, S. J., Hector, M. P. & Liversidge, H. M. Brief communication: the London atlas of human tooth development and eruption. *Am. J. Phys. Anthropol.* **142**, 481–490 (2010).

45. Harvig, L., Frei, K. M., Price, D. & Lynnerup, N. Strontium isotope signals in cremated petrous portions as indicator for childhood origin. *PLoS One* **9**, e101603 (2014).

46. Hillson, S. *Teeth* (Cambridge University Press, 2005), https://doi.org/10.1017/CBO9780511614477.

47. Snoeck, C. *et al.* Calcined bone provides a reliable substrate for strontium isotope ratios as shown by an enrichment experiment. *Rapid Communications in Mass Spectrometry* **29**, 107–114 (2015).

48. Bentley, R. A., Price, T. D. & Stephan, E. Determining the “local” ^87^Sr/^86^Sr range for archaeological skeletons: a case study from Neolithic Europe. *J. Archaeol. Sci.* **31**, 365–375 (2004).

49. Dasch, E. J. Strontium isotopes in weathering profiles, deep-sea sediments, and sedimentary rocks. *Geochim. Cosmochim. Acta* **33**, 1521–1552 (1969).

50. Toncala, A. *et al.* On the premises of mixing models to define local bioavailable ^87^Sr/^86^Sr ranges in archaeological contexts. *Science of The Total Environment* **745**, 140902 (2020).

51. Capo, R. C., Stewart, B. W. & Chadwick, O. A. Strontium isotopes as tracers of ecosystem processes: theory and methods. *Geoderma* **82**, 197–225 (1998).

52. Burton, J. H. & Hahn, R. Assessing the “local” ^87^Sr/^86^Sr ratio for humans in *Isotopic landscapes in bioarchaeology* (eds. Grupe, G. & McGlynn, G. C.) 113–121 (Springer Berlin Heidelberg, 2016), https://doi.org/10.1007/978-3-662-48339-8_6.

53. Oelze, V. M., Nehlich, O. & Richards, M. P. “There’s no place like home”: no isotopic evidence for mobility at the early Bronze Age Cemetery of Singen, Germany. *Archaeometry* **54**, 752–778 (2012).

54. Knipper, C. *et al.* Coalescing traditions – coalescing people: community formation in Pannonia after the decline of the Roman Empire. *PLoS One* **15**, e0231760 (2020).

55. Price, T. D., Wahl, J. & Bentley, A. Isotopic evidence for mobility and group organization among Neolithic farmers at Talheim, Germany, 5000 BC. *Eur J Archaeol* **9**, 259–284 (2006).

56. Wright, L. E. Identifying immigrants to Tikal, Guatemala: defining local variability in strontium isotope ratios of human tooth enamel. *J. Archaeol. Sci.* **32**, 555–566 (2005).

57. Lightfoot, E. & O’Connell, T. On the use of biomineral oxygen isotope data to identify human migrants in the archaeological record: intra-sample variation, statistical methods and geographical considerations. *PLoS One* **11**, e0153850 (2016).

58. Evans, J. A. & Tatham, S. Defining “local signature” in terms of Sr isotope composition using a tenth- to twelfth-century Anglo-Saxon population living on a Jurassic clay-carbonate terrain, Rutland, UK. *Geological Society, London, Special Publications* **232**, 237–248 (2004).

59. Alt, K. W. *et al.* Lombards on the move – an integrative study of the migration period cemetery at Szólád, Hungary. *PLoS One* **9**, e110793 (2014).

60. Knipper, C. *et al.* Female exogamy and gene pool diversification at the transition from the Final Neolithic to the Early Bronze Age in central Europe. *Proceedings of the National Academy of Sciences* **114**, 10083–10088 (2017).

61. Knipper, C. *et al.* A knot in a network: residential mobility at the Late Iron Age proto-urban centre of Basel-Gasfabrik (Switzerland) revealed by isotope analyses. *J. Archaeol. Sci. Rep* **17**, 735–753 (2018).

62. Alt, K. W. *et al.* Earliest evidence for social endogamy in the 9,000-year-old-population of Basta, Jordan. *PLoS One* **8**, e65649 (2013).

63. Díaz-Zorita Bonilla, M., Beck, J., Bocherens, H. & Díaz-del-Río, P. Isotopic evidence for mobility at large-scale human aggregations in Copper Age Iberia: the mega-site of Marroquíes. *Antiquity* **92**, 991–1007 (2018).

64. Lugli, F. *et al.* A strontium isoscape of Italy for provenance studies. *Chem. Geol.* **587**, 120624 (2022).

65. Miranda, P. Fermo (FM): la necropoli di contrada Mossa (Federico II di Napoli, 2019).

66. Esposito, C. La necropoli villanoviana di Fermo in località Misericordia. Scavi Brusadin (1956-1957) (Federico II di Napoli, 2015).

67. Montali, A. Fermo, Necropoli Misericordia: contributo per la conoscenza delle prime fasi. *Picus. Studi e ricerche sulle Marche nell’antichità* **26**, 183–261 (2006).

68. Miller, D. Stable carbon and nitrogen isotope analysis in Italy and Croatia: Bronze Age food practices across the Adriatic (Sapienza University of Rome, 2018).
